# *In vitro* characterization of solute transport in the spinal canal

**DOI:** 10.1063/5.0150158

**Published:** 2023-05-26

**Authors:** F. Moral-Pulido, J. I. Jiménez-González, C. Gutiérrez-Montes, W. Coenen, A. L. Sánchez, C. Martínez-Bazán

**Affiliations:** 1 Department of Mechanical and Mining Engineering, University of Jaén, 23071 Jaén, Spain; 2 Andalusian Institute for Earth System Research, University of Jaén, Campus de las Lagunillas, 23071 Jaén, Spain; 3 Department of Thermal and Fluid Engineering, University Carlos III of Madrid, 28911 Leganés, Spain; 4 Department of Aerospace and Mechanical Engineering, University of California, San Diego, California 92093-0411, USA; 5 Department of Mechanics of Structures and Hydraulic Engineering, University of Granada, 18001 Granada, Spain; 6 Andalusian Institute for Earth System Research, University of Granada, Avda. del Mediterráneo s/n, 18006 Granada, Spain

## Abstract

This paper presents results of an experimental investigation of solute transport in a simplified model of the spinal canal. The work aims to provide increased understanding of the mechanisms responsible for drug dispersion in intrathecal drug delivery (ITDD) procedures. The model consists of an annular channel bounded externally by a rigid transparent tube of circular section, representing the dura mater, and internally by an eccentric cylindrical compliant insert, representing the spinal cord. The tube, closed at one end, is connected to a rigid acrylic reservoir, representing the cranial cavity. The system is filled with water, whose properties are almost identical to those of the cerebrospinal fluid. A programmable peristaltic pump is employed to generate oscillatory motion at frequencies that are representative of those induced by the cardiac and respiratory cycles. Laser induced fluorescence is used to characterize the dispersion of fluorescent dye along the canal and into the cranial cavity for different values of the relevant Womersley number and different eccentricities of the annular section. The present work corroborates experimentally, for the first time, the existence of a steady bulk flow, associated with the mean Lagrangian motion, which plays a key role in the transport of the solute along the spinal canal. The measurements of solute dispersion are found to be in excellent agreement with theoretical predictions obtained using a simplified transport equation derived earlier on the basis of a two-timescale asymptotic analysis. The experimental results underscore the importance of the eccentricity and its variations along the canal and identifies changes in the flow topology associated with differences in the Womersley number, with potential implications in guiding future designs of ITDD protocols.

## I. INTRODUCTION

The cerebrospinal fluid (CSF) is an ultrafiltrate of plasma that bathes the entire surface of the central nervous system (CNS). It behaves as a Newtonian fluid with close-to-water physical properties (density $\rho =10^{3}\;\text{kg}/{\text{m}}^{3}$ and kinematic viscosity $\nu =0.71\times 10^{-6}{\;\text{m}}^{2}/\text{s}$ at body temperature). The total volume of CSF in a healthy adult human is around 140–170 ml, distributed between the cerebral ventricles (30 ml), the cerebral subarachnoid space (70–80 ml), and the spinal subarachnoid space (SSAS) (40–60 ml).^1^ CSF is mainly secreted in the choroid plexus via the ependymal cells that line the ventricles of the brain and is reabsorbed through the arachnoid villi, the total volume being renewed every 5 to 6 h.^2,3^ CSF acts as shock absorber for the brain. Besides, its presence induces a buoyancy force that effectively reduces the brain weight, thereby limiting the compression on the spinal-cord stem. In addition to these important mechanical functions, CSF has a number of physiological functions associated with the transport of hormones, nutrients, and neuroendocrine substances.^4,5^

**FIG. 1.**
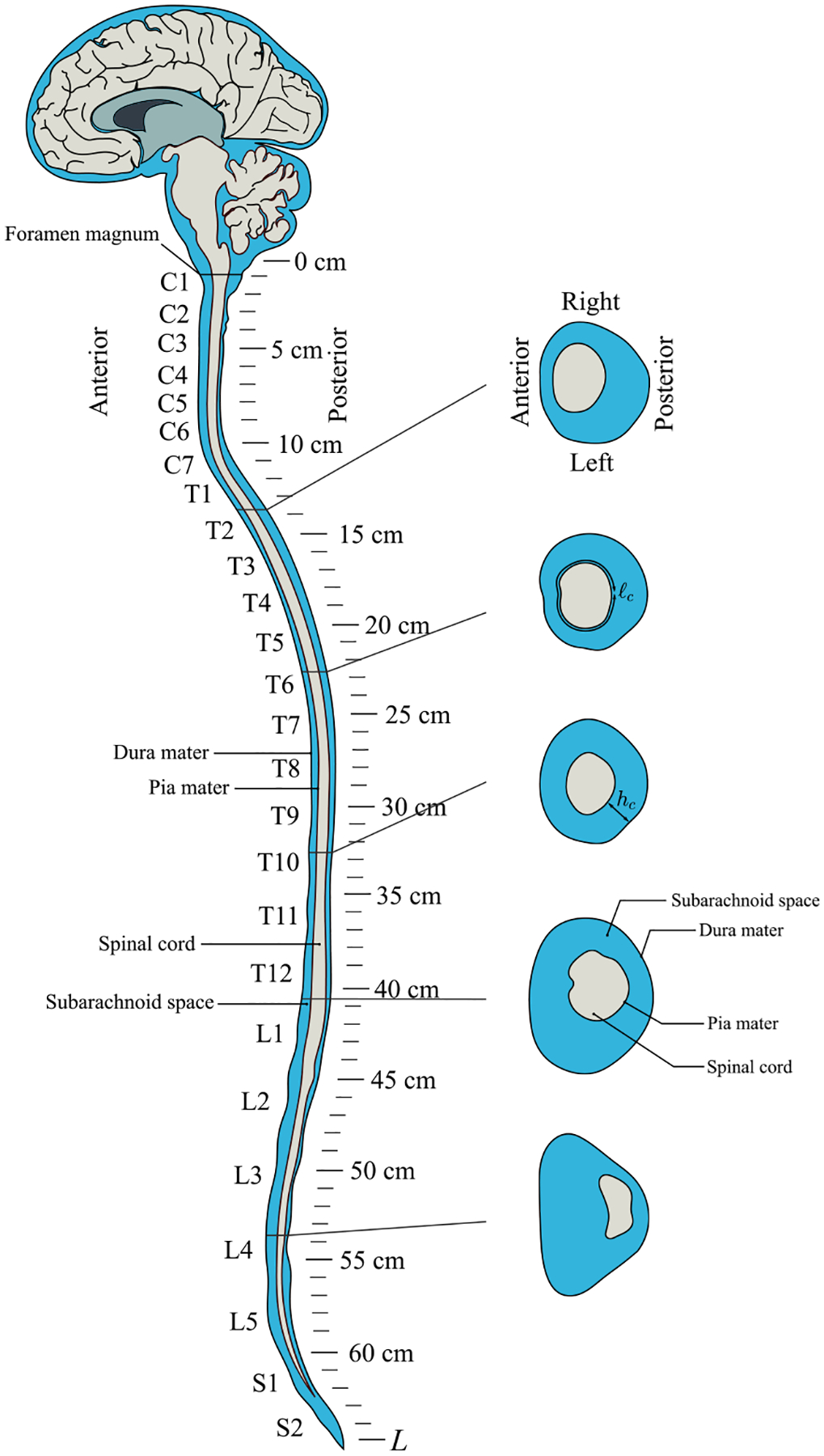
Anatomy of the spinal subarachnoid space of a healthy human with axial cuts at several locations and the CSF colored in blue.

Because of its importance in connection with physiological processes and its potential role in the development of neurological pathologies,^6,7^ the motion of CSF has been the subject of numerous theoretical, numerical, and *in vivo* and *in vitro* experimental studies (see, for example, the recent literature reviews given in Refs. 8 and 9). The focus of the present work will be on the motion occurring in the spinal subarachnoid space (SSAS), a slender, compliant, annular canal surrounding the spinal cord. As shown in Fig. 1, the spinal canal, which is connected to the cranial cavity through the foramen magnum and is closed at its distant sacral end, is bounded externally by the dura membrane, which separates it from an outer epidural layer containing fatty tissue and blood vessels, and internally by the pia membrane.

It is now well established that the CSF velocity in the SSAS, predominantly aligned with the spinal cord, displays pulsatile components synchronized with the cardiac and respiratory cycles, with peak values on the order of a few centimeters per second. The cardiac-driven motion, with a typical frequency of 1 Hz, is induced by pressure fluctuations in the cranial vault associated with the cyclic in-/outflow of arterial/venous blood.^10^ During each cardiac cycle, a small CSF volume ΔV≃1-2ml is pushed in and out of the spinal canal through the foramen magnum. This stroke volume is accommodated by the displacement of the dura and pia membranes, with the local CSF pressure fluctuations related to the local changes in the cross-sectional area of the SSAS through a complicated fluid–structure interaction problem involving the displacement of venous flow and fatty tissue.^9^ The cardiac-driven oscillatory flow rate is maximum near the foramen magnum and decays monotonically to zero at the sacrum, with typical peak values of order 5 ml/s in the upper cervical region and 1 ml/s in the lumbar region.^10,11^

Unlike the cardiac-driven motion, the flow component synchronized with the respiratory cycle,^12^ which exhibits frequencies on the order of 0.3 Hz, is not driven by the intracranial pressure fluctuations. Instead, its origin is hypothesized to lie in the pressure variations induced by respiration in the venous plexus, located in the epidural space of the lower thoracic and upper lumbar spine.^13^ Recent magnetic resonance (MR) measurements performed under normal breathing conditions have revealed that the associated flow rates are maximum near the thoracolumbar junction (1 – 3 ml/s) and much smaller in the cervical spine.^14^

Apart from the aforementioned purely oscillatory motion, CSF also undergoes a slow steady motion characterized by small velocities on the order of a centimeter per minute. Unlike the oscillatory flow, this “bulk motion” has the ability to transport solutes along the total distance of the spinal canal and therefore plays a key role in enabling the numerous physiological functions attributed to CSF. This bulk motion is also important in connection with the transport of drugs in intrathecal drug delivery (ITDD) procedures,^15^ a technique used to administer pain, analgesic, and cancer medication in which the drug is delivered directly into the CSF, typically through a lumbar puncture, thereby circumventing the blood–brain barrier.^16^ The widespread use of ITDD faces challenges related to underdosing and overdosing, with the former resulting in reduced therapeutic effects in the case of cancer treatments and the latter leading to permanent nerve damage in the case of pain medication.^17^ It is evident that a better understanding of the bulk motion of CSF is essential both to prevent physiological dysfunctions and pathologies of the CNS^6^ and to enable optimized subject-specific ITDD protocols.

While the existence of bulk motion has been known since the seminal radiographic observations of Di Chiro,^18^ its physical origin has been unveiled only relatively recently.^19^ The analysis considered a simplified model of the cardiac-driven oscillatory flow in agreement with the considerations described before. The associated Eulerian velocity field was computed using a perturbation analysis involving a small parameter ε representing the ratio ΔV/V≪1 of the stroke volume ΔV≃1-2ml to the total volume of CSF contained in the SSAS (V≃40-60ml). In the limit ε≪1, the velocity at the leading order was found to be purely oscillatory, with a zero time-averaged value. By way of contrast, the first-order corrections, associated with the nonlinear convective acceleration, were found to contain a steady-streaming component that corresponds to the bulk flow observed in *in vivo* experiments. The theory was applied to a simplified geometry, which consisted of an annular doubly slender canal, open at the entrance and closed at the end. (This steady component has also been studied with elliptical cross section geometries in recent works.^20^) Although complicating micro-anatomical features, such as nerve roots, dentriculate ligaments, and trabeculae, were not taken into account (see, for example, Refs. 10 and 21–27) the model did account for a key feature of the SSAS, namely, the eccentric placement of the spinal cord within the lumen of the spinal canal. The magnitude of the axial streaming flow was found to depend critically on the level of eccentricity.

The analysis of Ref. 19 was extended in Ref. 28 to show that the mean Lagrangian velocity experienced by a fluid particle in the spinal canal is the sum of the steady-streaming velocity, determined by time-averaging the Eulerian velocity field, and the so-called Stokes drift,^29^ a purely kinematic effect associated with the spatial nonuniformity of the pulsatile flow. One can understand the origin of the Stokes drift by noting that, in the presence of a velocity gradient, a fluid particle subject to an oscillating velocity field experiences during each oscillatory cycle an instantaneous velocity that differs by a small amount from that existing at the initial point at corresponding times. As a result, the fluid particle does not return to its original position at the end of the cycle. The Stokes drift arises as a result of the accumulation of displacements over subsequent cycles, yielding characteristic velocities that are comparable in magnitude to those of steady streaming. The asymptotic analysis performed in Ref. 28 also provided a reduced transport equation describing the dispersion of a solute carried by the CSF, with the mean Lagrangian velocity (i.e., the sum of the steady-streaming and Stokes-drift velocities) determining the convective transport rate in the long timescale characterizing dispersion along the spinal canal. The strength of the previous reduced models^19,28^ lies in the fact that they provide closed-form expressions for the time-averaged velocity field associated with the bulk motion, as well as simplified transport equations that describe the slow solute transport, which can be evaluated very efficiently, without the need to solve the flow over thousands of oscillation cycles, as required in direct numerical simulations targeting solute dispersion along the spinal canal.

As mentioned before, the eccentricity of the spinal canal has an important effect on the Lagrangian motion, and hence on the transport of solutes in the spinal canal. As shown in Fig. 1, in the human SSAS, this eccentricity changes longitudinally, as the spinal cord changes its position relative to the dura in the anteroposterior plane. Indeed, in healthy humans, the spinal canal exhibits concavity variations in the sagittal plane stemming from the four main curves of the spine, i.e., two kyphoses and two lordosis,^30^ characterized by the Cobb angles.^31^ In adults, the spinal cord located inside the spinal canal extends cranially from the brain to nearly the end of the L1 region.^30^ The spinal cord’s relative position inside the SSAS varies along the canal,^30,32^ as well as with posture,^33,34^ which yields subject-specific spinal canal eccentricity variations. In particular, the spinal cord is located near the posterior side of the canal in the cervical region, but close to the anterior side in most of the thoracic region, shifting posteriorly again as it approaches the lumbar region.^10,22,25,32,35^ This variable eccentricity has an important effect on the spatial structure of the Lagrangian motion, leading to the emergence of closed recirculating regions. These recirculating Lagrangian vortices have been computed on theoretical grounds for a realistic patient specific geometry^11^ and were corroborated in an idealized geometry by means of direct numerical simulations.^36^ The existence of these Lagrangian recirculating regions can have a strong clinical impact, since they modulate the rate of transport with which a drug injected intrathecally in the lumbar region is transported to the cranium. The presence of closed Lagrangian streamlines leads to augmented solute residence times in certain regions, thereby possibly increasing the risk of local overdose in ITDD procedures.

Beyond efforts to analytically and numerically model CSF flow and solute transport, a few attempts have recently been made to address the problem experimentally.^37–41^ In these works, different types of *in vitro* models, including an idealized channel with a simplified flow waveform^37^ and realistic patient-specific geometries with real CSF flow rates,^38,39^ have been considered, leading to promising results. However, these studies deal with rigid canals that are open at both ends and do not directly address the role of the compliant dura membrane and the associated fluid–structure interaction problem, an important aspect of the CSF dynamics in the SSAS.

Motivated by the clinical relevance of solute transport in the spinal canal and the lack of a thorough experimental characterization of the problem, we present here an *in vitro* experimental study of the solute transport along a compliant spinal canal. We begin by giving in Sec. II a description of the experimental facility and experimental techniques employed in this study. The facility is designed according to the models discussed in our previous works,^19,28,36^ with consideration given to the case in which the annular canal has constant eccentricity and the more realistic case in which the eccentricity varies with the distance from its entrance. A brief description of the analytical flow and transport models developed earlier^19,28^ is provided in Sec. III. The experiments, reported in Sec. IV, are used to investigate effects of eccentricity and oscillating frequency. The experimental results are supported by predictions given by the analytical model. Finally, conclusions are drawn in Sec. V.

## II. EXPERIMENTAL FACILITY AND EXPERIMENTAL TECHNIQUES

**FIG. 2.**
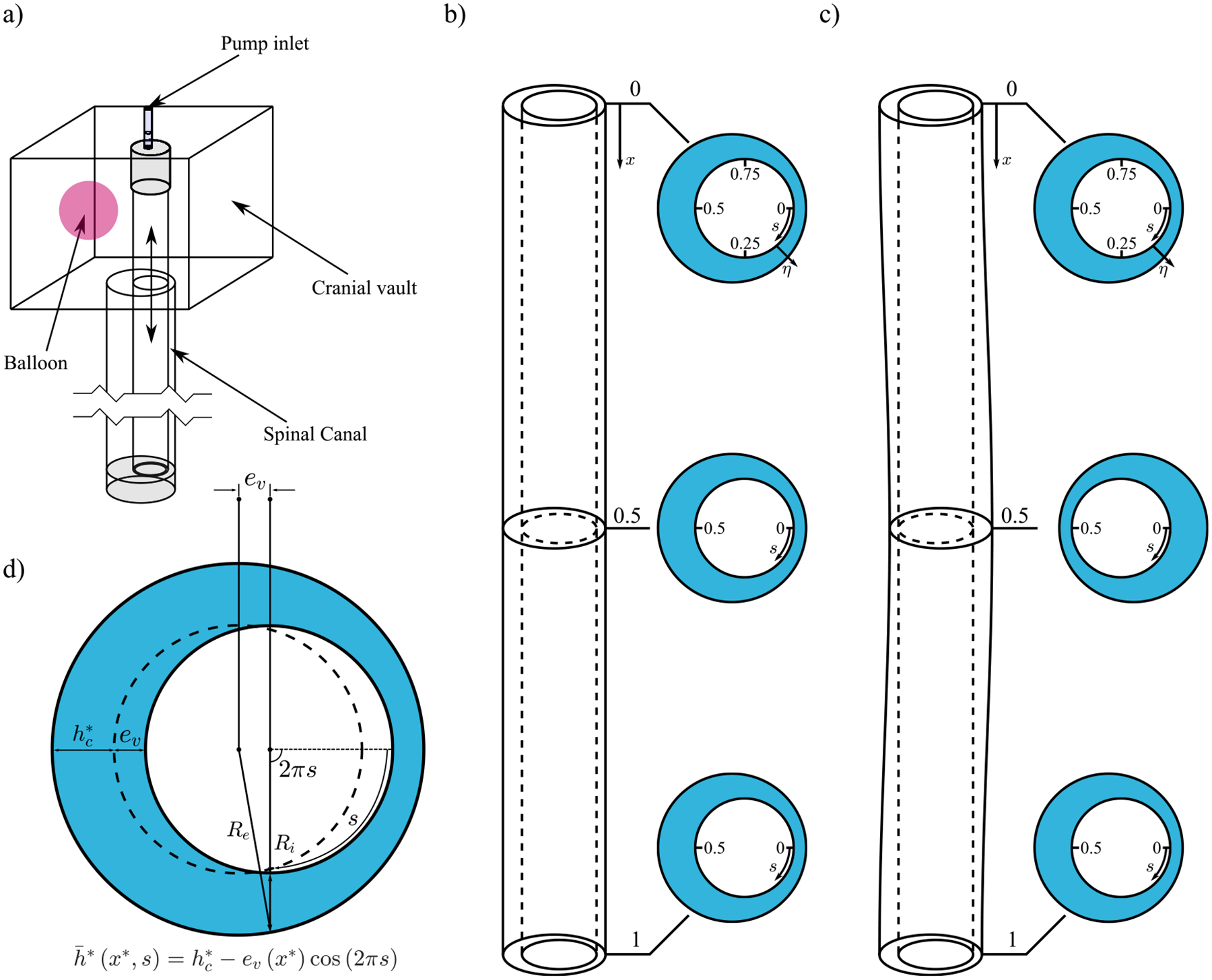
(a) Schematic representation of the experimental *in vitro* facility of the SSAS. (b) Representation of the constant eccentricity canal and (c) variable eccentricity canal, with cross sections at planes $x=x^{*}/L=0,0.5,\text{and}\;1$, showing the dimensionless curvilinear coordinates (x,s,η), which vary from zero to unity, defined in Sec. III. (d) Schematic view of the unperturbed canal width and the eccentricity, $e_{v}$, where $e_{v}\left(x^{*}\right)=e$ in the constant eccentricity canal and $e_{v}\left(x^{*}\right)=e\;\text{cos}\left(2\pi x^{*}/L\right)$ in the variable one.

The experimental facility used in our study, to be described below, enables the quantification of effects of spinal-cord eccentricity and flow frequency on solute transport in the spinal canal. The experiments were performed in an *in vitro* model of the subarachnoid space, using distilled water of density $\rho =998.2\;\text{kg}/{\text{m}}^{3}$ and kinematic viscosity $\nu =10^{-6}{\;\text{m}}^{2}/\text{s}$ as the working fluid. The model consisted of a 15 × 15 × 15 cm^3^ acrylic tank, emulating the cranial vault, connected to a plexiglass tube of length L=50cm, representing the dura mater. To allow for temporal variations of the local cross-sectional area, needed to accommodate the oscillating flow, a hollow flexible tube of circular section was placed inside, with its distal end anchored at the closed bottom of the rigid tube and its proximal end connected to a peristaltic pump, as indicated in Fig. 2(a). The flexible tube, with outer radius $R_{i}=7\;\text{mm}$ and thickness $h_{s}=2\;\text{mm}$, was made up of rubber (Shore hardness 60 A, elastic modulus of approximately $E_{c}=2.2\;\text{MPa}$, and tensile strength of $T_{s}=11\;\text{MPa}$), yielding an elastic wave of characteristic wavelength ${\left(E_{c}/\rho \right)}^{1/2}/\omega$, larger than the tube length L. The deformation of the inner tube associated with this elastic wave drives the motion in the annular canal. The expressions for the local variation of the cross-sectional area can be derived, as done in Ref. 36, enabling the elastic wave to be related to the pressure variations along the canal. Note that the speed of the elastic wave in the experiments ${\left(E_{c}/\rho \right)}^{1/2}≃45\;\text{m}/\text{s}$ is comparable to, although somewhat larger than, those reported in the literature, with values ranging from 3.5 to 33.8 m/s for subjects under different conditions.^42–45^

To explore effects of spinal-cord eccentricity, two different canal geometries were implemented, as indicated in Fig. 2. Most experiments considered the geometry depicted in Fig. 2(b), in which the outer tube is a circular cylinder of inner radius $R_{e}=10.5\;\text{mm}$ that lies parallel to the inner tube, so that the resulting canal eccentricity, characterized by the distance between their axes, e, remains constant along the canal. The eccentricity can be changed using cams with different eccentricities in the lower and upper parts of the facility, where the flexible tube was anchored. In this case, the resulting undeformed canal width can be approximated by the expression ${\overset{‾}{h}}^{*}(s)=h_{c}^{*}[1-\beta \;\text{cos}(2\pi s)]$, where $h_{c}^{*}=\left(R_{e}-R_{i}\right)=3.5\;\text{mm}$ is the average canal width, $\beta =e/h_{c}^{*}<1$ is the dimensionless eccentricity, and s is the azimuthal distance normalized with the perimeter of the inner tube $\ell^{*}=2\pi R_{i}$, with 0≤s≤1 and 2πs being the corresponding azimuthal angle [see Fig. 2(d)]. A limited set of experiments used the geometrical configuration shown in Fig. 2(c), involving an outer rigid tube with longitudinal curvature, resulting in a canal eccentricity that varies with the distance from the canal entrance $x^{*}$ according to $e_{v}=e\;\text{cos}\left(2\pi x^{*}/L\right)\;\text{mm}$, where e=1.5mm was fixed. In this case, the circular section of the outer tube has inner radius $R_{e}=10\;\text{mm}$, so that $h_{c}^{*}=3\;\text{mm}$ and $\beta =e/h_{c}^{*}=0.5$. In this case, the canal width varies with both $x^{*}$ and s according to ${\overset{‾}{h}}^{*}=h_{c}^{*}\left[1-\beta \;\text{cos}\left(2\pi x^{*}/L\right)\;\text{cos}(2\pi s)\right]$. A programmable peristaltic pump was used to generate a flow rate varying harmonically with time $t^{*}$ according to $Q\left(t^{*}\right)=Q_{max}\;\text{sin}\left(\omega t^{*}\right)$ of amplitude $Q_{\text{max}}$ and angular frequency ω, the latter related to the period T and frequency f by ω=2πf=2π/T. The stroke volume that enters and leaves the flexible tube during each cycle, given by $\Delta V=\int_{0}^{\pi /\omega }Q\;\text{d}t^{*}=2Q_{max}/\omega$, was chosen to be a small fraction of the volume contained in the annular canal $V=\pi L\left(R_{e}^{2}-R_{i}^{2}\right)$, resulting in values of ΔV/V≈0.018-0.084 similar to those observed in the SSAS.^9^ The volume changes induced by the expansions and contractions of the flexible tube were accommodated in the facility thanks to the presence of a compressible air balloon placed inside the tank [see Fig. 2(a)].

**FIG. 3.**
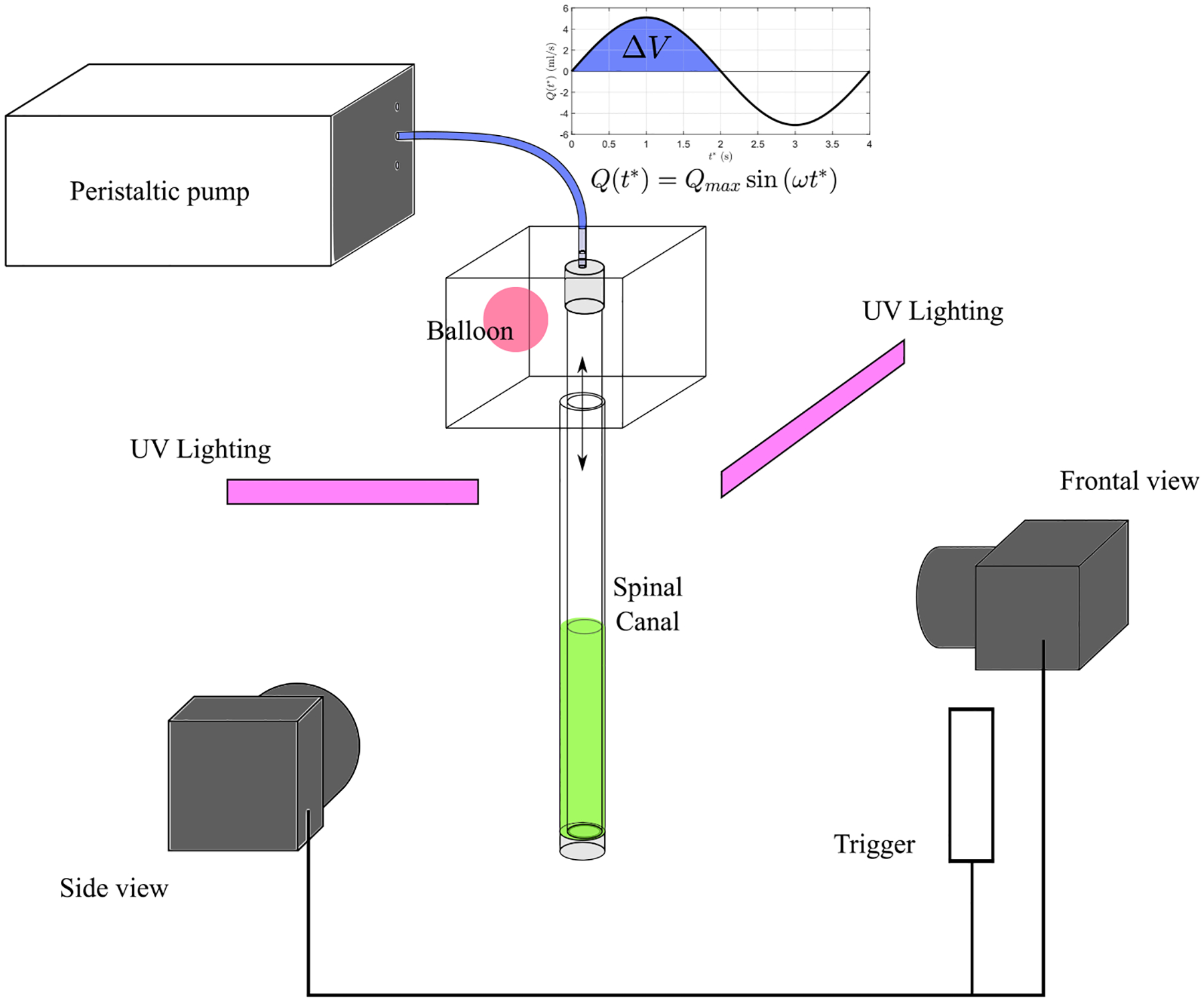
Schematic view of the complete setup: *in vitro* model of the subarachnoid space, cameras, programmable peristaltic pump, and UV lighting. The flow rate wave form provided by the pump is also displayed in the figure with $Q\left(t^{*}\right)=Q_{max}\;\text{sin}\left(\omega t^{*}\right)$, and the corresponding stroke volume, $\Delta V=2Q_{max}/\omega$.

Two types of experiments were performed using laser induced fluorescence techniques (LIF). The first one was used to determine the transport of a solute concentration along the spinal canal, from an initial position $x_{0}^{*}/L=0.5$ from the entrance of the canal. In these experiments, the bottom half of the canal (from $x^{*}/L=0.5$ to $x^{*}/L=1$) was carefully filled with fluorescent dye of sodic fluorescein, of diffusivity $\kappa \approx 4\times 10^{-10}{\;\text{m}}^{2}/\text{s}$ and corresponding Schmidt number S=ν/κ≈2500, at a concentration $C=1.6\;\text{g}/{\text{cm}}^{3}$ using a syringe pump. The facility was illuminated with UV LED’s lights, and the ascending motion of the solute, induced by the peristaltic pump, was recorded with two synchronized reflex cameras, placed at two perpendicular planes, i.e., s=0 and s=0.25 (see Fig. 3). During the experiments, the sampling period was adjusted to lie between two and eight times the period of the oscillating flow to assure a minimum of 200 images per test. Thus, we were able to track the front of the filament moving toward the upper reservoir until it reached the canal entrance. The second type of experiments focused on the evolution of the solute concentration in the cranial vault, an important aspect for ITDD procedures targeting brain tumors. In these experiments, the tank was illuminated with a black light and the time evolution of the intensity of the light emitted by the fluorescein reaching the tank was recorded with a CCD camera, with the sampling period selected to be 30 times the oscillatory period T. Since the light intensity is proportional to the solute concentration, these measurements provided a useful quantification of the time evolution of the concentration of solute reaching the cranial vault.

For the two types of experiments described above, the images were processed with a custom MATLAB^®^ routine as described below. First, a background image was subtracted from all the images to eliminate the external noise induced by the UV-light. Since the wavelength of light emitted by the fluorescein is λ=541nm, corresponding to green color, only the green component of the RGB image was processed and converted to a gray-scale image. Afterward, the minimum value of the intensity of each pixel of the images recorded was subtracted from all the images to enhance the solute detection process and to avoid the generation of shadows and bright areas, which could be wrongly interpreted as a part of the solute motion. The preprocessed images were subsequently post-processed to determine the motion of the front of the tracer through the application of an image analysis routine based on that developed by Refs. 38, 39, and 46. For the second type of experiments, measuring the temporal evolution of the solute concentration in the cranial vault, two windows, located on both sides of the flexible tube crossing the reservoir, were selected and the time evolution of the averaged value of the tracer concentration in each region was determined. To be able to compare the results, all the experiments were performed under the same conditions of light intensity, UV LED light location, and initial solute concentration. This analysis allowed us to obtain not only the time taken for the solute to reach the cranial vault, but also the amount of solute entering it.

A large number of experiments were performed using the straight configuration depicted in Fig. 2(b) for different values of the eccentricity β and the oscillating frequency ω=2πf, the latter characterized in the following plots by the associated Womersley number $\alpha ={\left(h_{c}^{*2}\omega /\nu \right)}^{1/2}$ (see Table I). To explore the effects of canal eccentricity on solute transport, three values of β were tested, namely, β=0.14,0.28,and0.42, using the straight configuration with α=4.39. Experiments 1 to 6 in Table I were devoted to the description of the time evolution of the tracer front along the canal, while experiments 7 to 12 focused on the description of the time evolution of the concentration of solute in the reservoir (cranial vault). In addition, to study the effect of the oscillation frequency, a series of experiments were performed varying α from 3.04 to 11.77 (experiments 13 to 16), for β=0.42. The variable-eccentricity configuration of Fig. 2(c) was used in experiments 17 and 18 for a fixed value of the Womersley number α=4.45. In particular, experiment 17 focuses on the motion of the tracer along the curved SSAS, while experiment 18 aims at describing the evolution of the tracer concentration in the cranial vault.

## III. MATHEMATICAL MODEL

The flow of CSF in the spinal canal fundamentally involves a fluid–structure interaction problem governed by the Navier–Stokes equations for an incompressible fluid, together with a constitutive law characterizing the deformable dura membrane behavior. In addition, to describe the transport of a solute of molecular diffusivity κ, the species transport equation should also be solved.^36^ In our previous works,^19,28^ the flow and transport of the CSF along the SSAS was described in terms of dimensionless curvilinear coordinates, including the normalized axial and azimuthal coordinates $x=x^{*}/L$ and s introduced above along with the transverse coordinate η, defined as the transverse distance to the inner surface normalized with the local width, $h^{*}(x,s,t)$, so that all coordinates vary from zero to unity (see Fig. 2). Here, $t=t^{*}\omega$ represents the dimensionless time, and the variables with asterisks denote dimensional variables. Since a detailed derivation of the reduced-order equations is available in previous publications,^19,28^ we shall only give below a succinct description of the model.

In the analysis, the cranial pressure oscillations are assumed to be harmonic, described by $\left(\Delta p{)}_{c}\;\text{cos}(t\right)$, with $(\Delta p{)}_{c}$ representing the intracranial pressure fluctuation amplitude. Since the canal is slender, in that the characteristic values of the canal length L, spinal-cord perimeter $\ell_{c}^{*}$, and characteristic SSAS width $h_{c}^{*}$ satisfy $L\gg \ell_{c}^{*}\gg h_{c}^{*}$, terms of order ${\left(\ell_{c}^{*}/L\right)}^{2}$ and ${\left(h_{c}^{*}/L\right)}^{2}$ (and smaller) can be neglected in the conservation equations, as well as those associated with the small curvature along the spinal canal (see Ref. 19). The small local deformations $\delta h^{*}$ of the canal width $h^{*}={\overset{‾}{h}}^{*}+\delta h^{*}$, induced by the local pressure variations $\delta p^{*}$, are described using a linear elastic model $\delta h^{*}=\gamma^{*}\delta p^{*}$, where $\gamma^{*}$ measures the canal compliance,^10,28,36^ spatial variations of that are accounted for by introduction of the dimensionless function $\gamma (x,s)=\gamma^{*}/\gamma_{c}^{*}$, where $\gamma_{c}^{*}$ is the characteristic value of $\gamma^{*}$. The canal compliance is limited, in that

(1) $$\varepsilon =\frac{\gamma_{c}^{*}(\Delta p{)}_{c}}{h_{c}^{*}}≃\frac{\Delta V}{V}\approx \frac{\Delta L}{L}\ll 1,$$

where ΔL is the characteristic value of the stroke length.

An order-of-magnitude of the Navier–Stokes equations shows that the convective acceleration, of order $u_{c}^{2}/L$, is ε smaller than the local acceleration, of order $\omega u_{c}$, where $u_{c}\sim \omega \Delta L\sim \varepsilon \omega L$ is the characteristic axial flow velocity. The viscous force per unit mass, of order $u_{c}\nu /h_{c}^{*2}$, scales in the dimensionless formulation with the inverse of $\alpha^{2}$, where

(2) $$\alpha ={\left(\frac{h_{c}^{*2}\omega }{\nu }\right)}^{1/2}$$

is the relevant Womersley number, typically in the range of 3≲α≲10. On the other hand, the deformable behavior of the dura membrane is characterized by the dimensionless wavenumber

(3) $$k=\frac{\omega L}{{\left[\left(h_{c}^{*2}\gamma_{c}^{*}\right)/\rho \right]}^{1/2}},$$

representing the ratio canal length to the characteristic wavelength of the elastic wave.

In the dimensionless formulation,^19,28^ the geometry of the canal is characterized by the dimensionless functions $\ell (x)=\ell^{*}/\ell_{c}^{*}$ and $h(x,s,t)=h^{*}/h_{c}^{*}$. The limited compliance of the dura membrane leads to small changes of the canal width $h(x,s,t)=\overset{‾}{h}(x,s)+\varepsilon h^{\prime }(x,s,t)$, where $\overset{‾}{h}(x,s)$ is the unperturbed canal width, and $h^{\prime }(x,s,t)$ represents the time-dependent radial deformation. The streamwise, azimuthal, and traverse components of the velocity were scaled with their corresponding characteristic values, namely, $u_{c}=\varepsilon \omega L,w_{c}=\varepsilon \omega \ell_{c}^{*}$, and $v_{c}=\varepsilon \omega h_{c}^{*}$, respectively, to give (u,w,v). In addition, the streamwise pressure difference from the entrance value was scaled with its characteristic value $\rho \varepsilon \omega^{2}L^{2}$, whereas the small pressure variations around the canal at a fixed value of x, which are necessary to describe the azimuthal motion, are scaled with its corresponding characteristic value $\rho \varepsilon \omega^{2}\ell_{c}^{*2}$ to give the functions $p^{\prime }(x,t)$ and $\overset{ˆ}{p}(x,s,t)$, respectively.

The problem can be solved under the assumptions of canal slenderness $L\gg \ell_{c}^{*}\gg h_{c}^{*}$ and small compliance ε≪1, by introducing regular asymptotic expansions in powers of ε for all variables (i.e., $\phi =\phi_{0}+\varepsilon \phi_{1}+\varepsilon^{2}\phi_{2}+\cdots$, with ϕ representing any unknown function). The nonlinear terms associated with convective acceleration, of order ε, can be neglected at the leading order, leading to a linear unsteady lubrication problem involving a linear elastic law, the solution of which depends on the specific geometry of the SSAS through the functions ℓ(x) and h(x,s,t), on the dimensionless elastic-wave number k and the Womersley number, α and on the compliance distribution, measured by the function γ(x,s). The resulting velocity components and wall deformation can be expressed in the harmonic form $u_{0}=\text{Re}\left(iUe^{it}\right),w_{0}=\text{Re}\left(iWe^{it}\right),v_{0}=\text{Re}\left(iVe^{it}\right)$, and $h_{0}^{\prime }=\text{Re}\left(H^{\prime }e^{it}\right)$, where U,W,V, and $H^{\prime }$ are complex functions carrying the spatial dependence. Since at the leading order, the time-averaged values are zero, it is the first-order corrections, arising from the nonlinear effects associated with the convective acceleration and the canal deformation, that induce a nonzero steady component $\left(u_{SS},w_{SS},v_{SS}\right)$. This steady-streaming motion^47^ has longitudinal velocities that are of order $\varepsilon^{2}\omega L$, resulting in residence times in the spinal canal of order $\varepsilon^{-2}\omega^{-1}\gg \omega^{-1}$. Since ε~1/40, it follows that the residence time is about half an hour, to be compared with the period of the cardiac cycle (i.e., T≃1s).

As shown in Ref. 28, the mean Lagrangian motion following a fluid particle has an additional component arising from the so-called Stokes drift, whose magnitude is comparable to that of the steady-streaming velocities, so that the mean Lagrangian velocity determining the slow convective transport of the solute in the spinal canal is given by $\left(u_{L},v_{L},w_{L}\right)=\left(u_{SS}+u_{SD},v_{SS}+v_{SD},w_{SS}+w_{SD}\right)$. The dispersion of the solute includes an additional diffusion contribution that scales with the solute molecular diffusivity κ. The disparity of times scales between the oscillatory motion of CSF particles, with characteristic time $\omega^{-1}$, and the time-averaged Lagrangian motion, with characteristic time $\varepsilon^{-2}\omega^{-1}$, enables a two-timescale asymptotic analysis to be performed, leading to a reduced transport equation

(4) $$\frac{\partial C}{\partial \tau }+u_{\text{L}}\left(\frac{\partial C}{\partial x}-\frac{\partial \overset{‾}{h}}{\partial x}\frac{\eta }{\overset{‾}{h}}\frac{\partial C}{\partial \eta }\right)+\frac{v_{\text{L}}}{\overset{‾}{h}}\frac{\partial C}{\partial \eta }+\frac{w_{\text{L}}}{\ell }\left(\frac{\partial C}{\partial s}-\frac{\partial \overset{‾}{h}}{\partial s}\frac{\eta }{\overset{‾}{h}}\frac{\partial C}{\partial \eta }\right)=\frac{1}{\alpha^{2}\varepsilon^{2}S{\overset{‾}{h}}^{2}}\frac{\partial^{2}C}{\partial \eta^{2}},$$

for the solute concentration C(x,s,η,τ) involving the slow time variable $\tau =\varepsilon^{2}t$ and the Schmidt number $S=\nu /\kappa \sim \varepsilon^{-2}\gg 1$. Note that, since Eq. (4) only accounts for transverse molecular diffusion, the streamwise dispersion of the solute is driven entirely by convective transport. The above simplified description, whose accuracy was tested in Ref. 36 via comparisons with the results of direct numerical simulations, will be used below for generating theoretical predictions, to be compared with the experimental results.

## IV. RESULTS

The effects of eccentricity and pulsation frequency on the dispersion of a solute are to be investigated below using the *in vitro* experiments and mathematical model described above. We begin by presenting in Sec. IV A the experimental results obtained with the constant-eccentricity model depicted in Fig. 2(b) for values of β ranging from β=0.14 to β=0.42 and Womersley numbers ranging from α=3.04 to α=11.77. The configuration with variable eccentricity shown in Fig. 2(c) is investigated separately in Sec. IV B for a Womersley number equal to α=4.45. The measurements will be compared with predictions from the theoretical analysis, which will also be used to describe the velocity field along the canal.

Attention will be focused on the evolution of the solute front. In particular, the temporal variation of the front shape and its advance rate along the canal toward the cranial vault will be registered and compared with predictions obtained from the model with S≈2500. In presenting the results, the axial coordinate and the time will be expressed in the dimensionless form $x=x^{*}/L$ and $\tau =\varepsilon^{2}\omega t^{*}=\varepsilon^{2}t$, respectively, as consistent with the theoretical model.^28^

### A. Constant eccentricity configuration: Effect of β and α

**FIG. 4.**
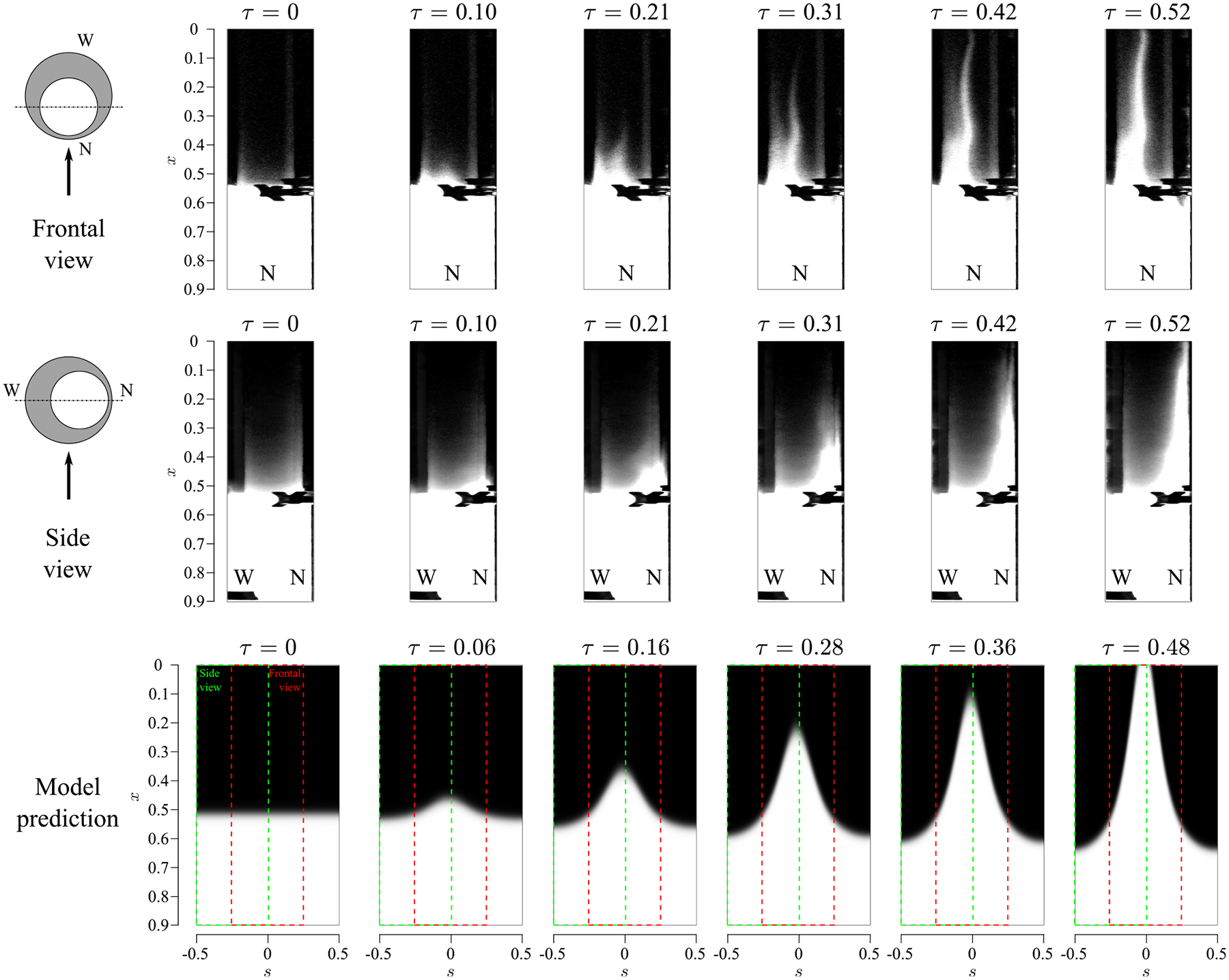
Time sequence of the experimental evolution of the solute along the spinal canal for β=0.42 and α=4.39 (experimental set 5 in Table I). The upper and central rows show the experimental frontal and side views, while the bottom row shows the results obtained from the mathematical model. (The red and green boxes represent the frontal and side views, respectively.) Here, W and N indicate the widest and the narrowest sections of the canal.

Let us begin by analyzing the case corresponding to β=0.42 and α=4.39, as the reference case. Figure 4 shows a sequence of snapshots of the temporal evolution of a solute initially filling the tube from x=0.5 to x=1. The frontal view shows the projection on a vertical plane at s=0.25, where the narrowest section is at the central axis of the view, while the lateral view shows a projection on a vertical plane at s=0, where the widest section is on the left of the image and the narrowest one on the right. Note that, since the experimental images are in fact projections of a three-dimensional view, the abscissa axes do not really show the variable s. The inner, compliant tube can also be seen in the images and should not be confused with the light emitted by the fluorescein. Although perturbed, the ascending motion of the solute is nearly symmetric with respect to the symmetry plane around the narrowest section, as observed in the frontal view. For this configuration, the solute is observed to move upward (cranial direction) along a small region within the narrowest part of the canal, s=0, whereas it moves toward the distal end (caudal direction) around the widest part of the canal, s=0.5. The decrease in the area close to the widest region is not appreciated in the images because, as previously stated, they show the projection on a plane of a three-dimensional view. Thus, the liquid in front of the narrowest section, whose level is higher, blocks the view of that area. Furthermore, it can be inferred from the time sequences that the front advances with a speed that increases as it gets closer to the cranial vault, as it will be later corroborated. The model predictions show reasonably good agreement with the measurements. In particular, as displayed in the bottom panel of Fig. 4, the solute exhibits cranial motion along the narrowest region and descends toward the distal end along the widest region. In addition, the transport velocity is also seen to increase near the canal entrance. The quantitative agreement between the experiments and the model is excellent, as seen by comparing the time needed for the solute to reach the canal entrance as predicted by the model (τ=0.48) and by the experiments (τ≈0.52).

**FIG. 5.**
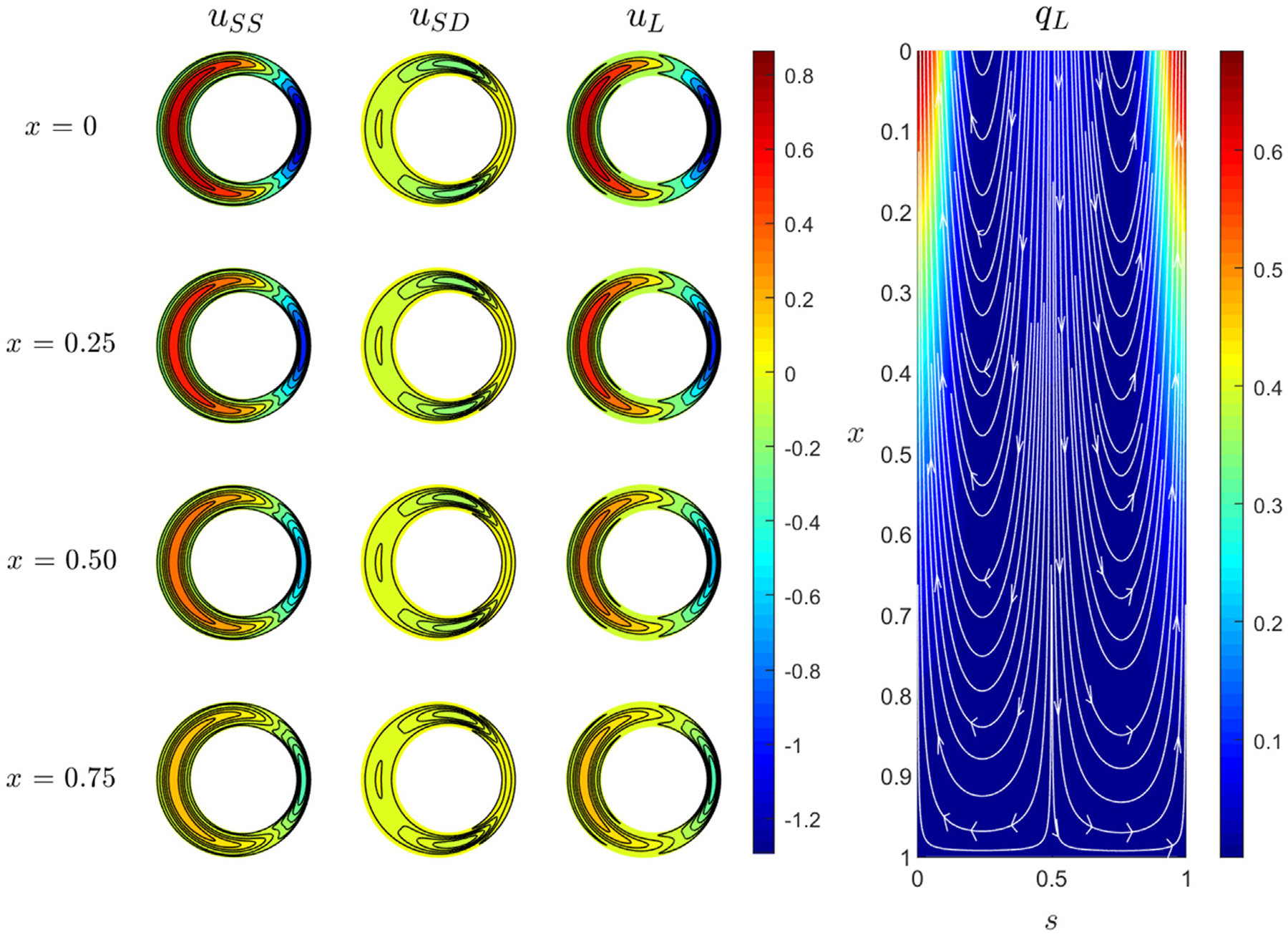
Distribution of streamwise components of the steady-streaming, Stokes-drift, and Lagrangian velocity fields at different sections x in the first, second, and third column, respectively. Streamlines obtained for the width-averaged Lagrangian components $\int_{0}^{1}u_{L}d\eta$ and $\int_{0}^{1}w_{L}d\eta$ with distribution of width-averaged Lagrangian velocity magnitude, $q_{L}={\left[{\left(\int_{0}^{1}u_{L}d\eta \right)}^{2}+{\left(\int_{0}^{1}w_{L}d\eta \right)}^{2}\right]}^{0.5}$.

The mathematical model will be used to analyze in detail the experimental results shown in Fig. 4. Unless otherwise stated, the computations assume a uniform compliance factor γ=1. Figure 5 shows distributions of the streamwise components of the steady-streaming, $u_{SS}$, Stokes-drift, $u_{SD}$, and Lagrangian velocities, $u_{L}$, respectively, at different distances from the entrance, x. As it can be observed, the steady streaming motion is directed toward the distal end around s=0.5 (widest section) and toward the canal entrance close to s=0 (narrowest section), whereas the Stokes drift shows a downward movement along s=0 and upward along s=0.5, the latter becoming larger for intermediate values of s and peaking close to s=±0.25. The steady-streaming velocity is significantly larger than the Stokes-drift velocity, so that the resulting distribution of Lagrangian velocity, shown in the third column of Fig. 5, is similar to that of steady streaming, shown in the first column. The associated induced net convective flow can be characterized by representing the Lagrangian streamlines of the width-averaged values of the axial and azimuthal velocities $\int_{0}^{1}u_{L}d\eta$ and $\int_{0}^{1}w_{L}d\eta$ in a s-x plane (see Fig. 5). A net stationary motion is observed entering the channel and moving down through s=0.5 at a relatively large velocity, as indicated by the proximity of the streamlines. The motion decelerates with the distance from the canal entrance, where the fluid moves with an azimuthal component toward s=0, and begins to rise toward the cranial vault with a speed that increases as it approaches the entrance of the canal (i.e., as x decreases).

**FIG. 6.**
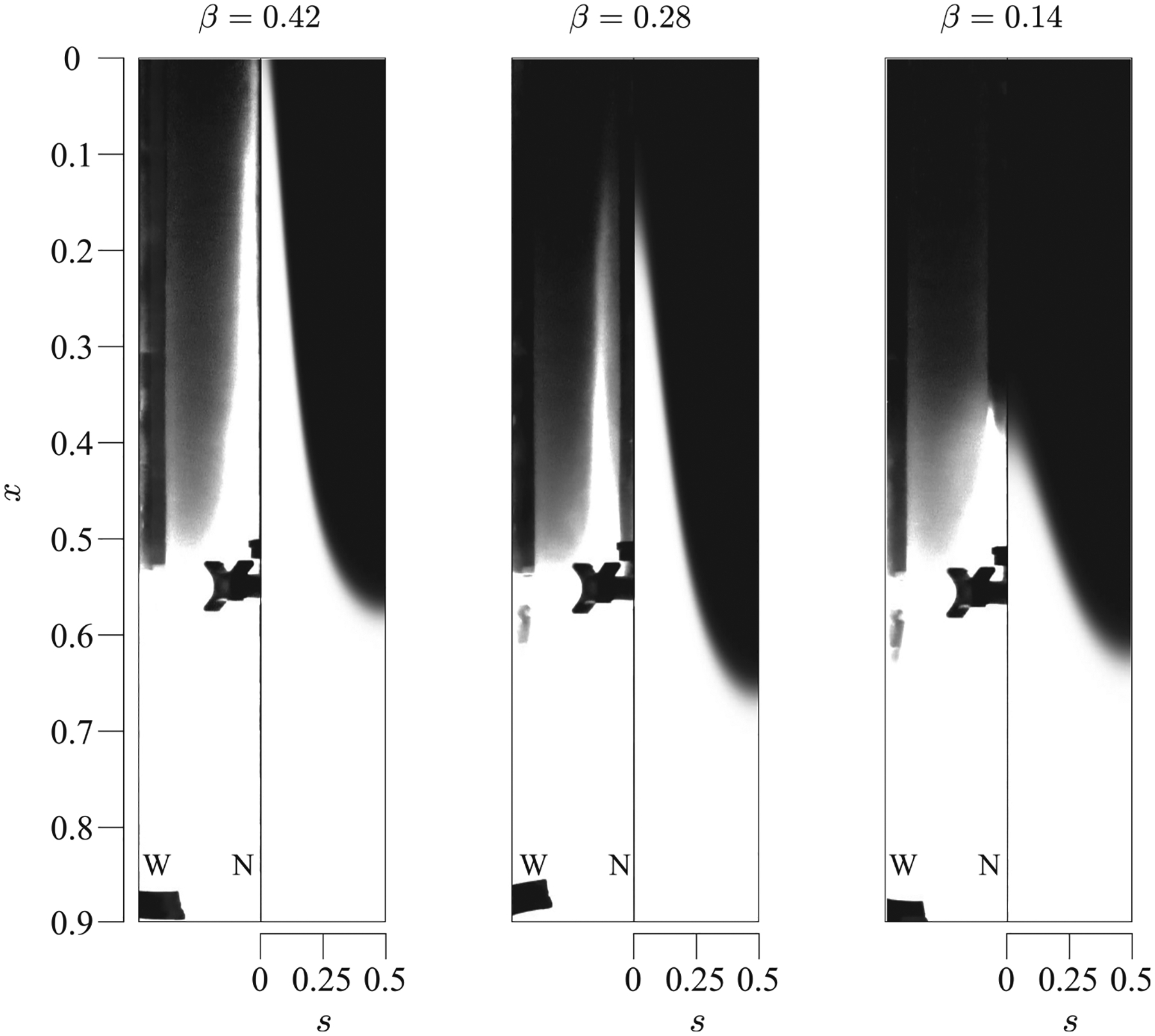
Distribution of a solute, initially filling the canal from x=0.5 to x=1, for β=0.42,β=0.28, and β=0.14, at τ=0.48 in the straight configuration, corresponding to experiments 5, 3, and 1 of Table I, respectively. Experimental results are represented on the left-hand side of the panels, while the theoretical predictions obtained integrating Eq. (4) are represented on the right-hand side. Here, N and W indicate the location of the narrowest and widest sections in the experimental visualizations.

The effect of the eccentricity on the transport of a solute was further investigated experimentally by varying the distance between the axes of both cylinders, e, to yield values of β equal to 0.14, 0.28, and 0.42, respectively (see Fig. 2), while maintaining a constant value of the Womersley number α=4.39. Snapshots at τ=0.48 are shown in Fig. 6 for the three values of β and compared with the results obtained from the theoretical model. Two volume ratios ΔV/V were considered, namely, 0.052 and 0.084 (Table I), yielding similar results. From the measurements, it can be observed that, while β hardly affects the flow topology in the range considered, i.e., the solute travels upward around s=0 and downward around s=0.5, it has a major impact on the net flow velocity and, thus, the time needed for the solute to reach the cranial vault. As seen in the snapshots for τ=0.48, increasing the eccentricity results in larger transport velocities, so that the front of the solute distribution, initially located at x=0.5, reaches x≈0.35 for β=0.14,x≈0.12 for β=0.28, and x≃0 for β=0.42. The right-hand side of each panel in Fig. 6 shows the corresponding predictions of the solute distribution given by the theoretical model, yielding results in reasonably good agreement with the experimental observations.

**FIG. 7.**
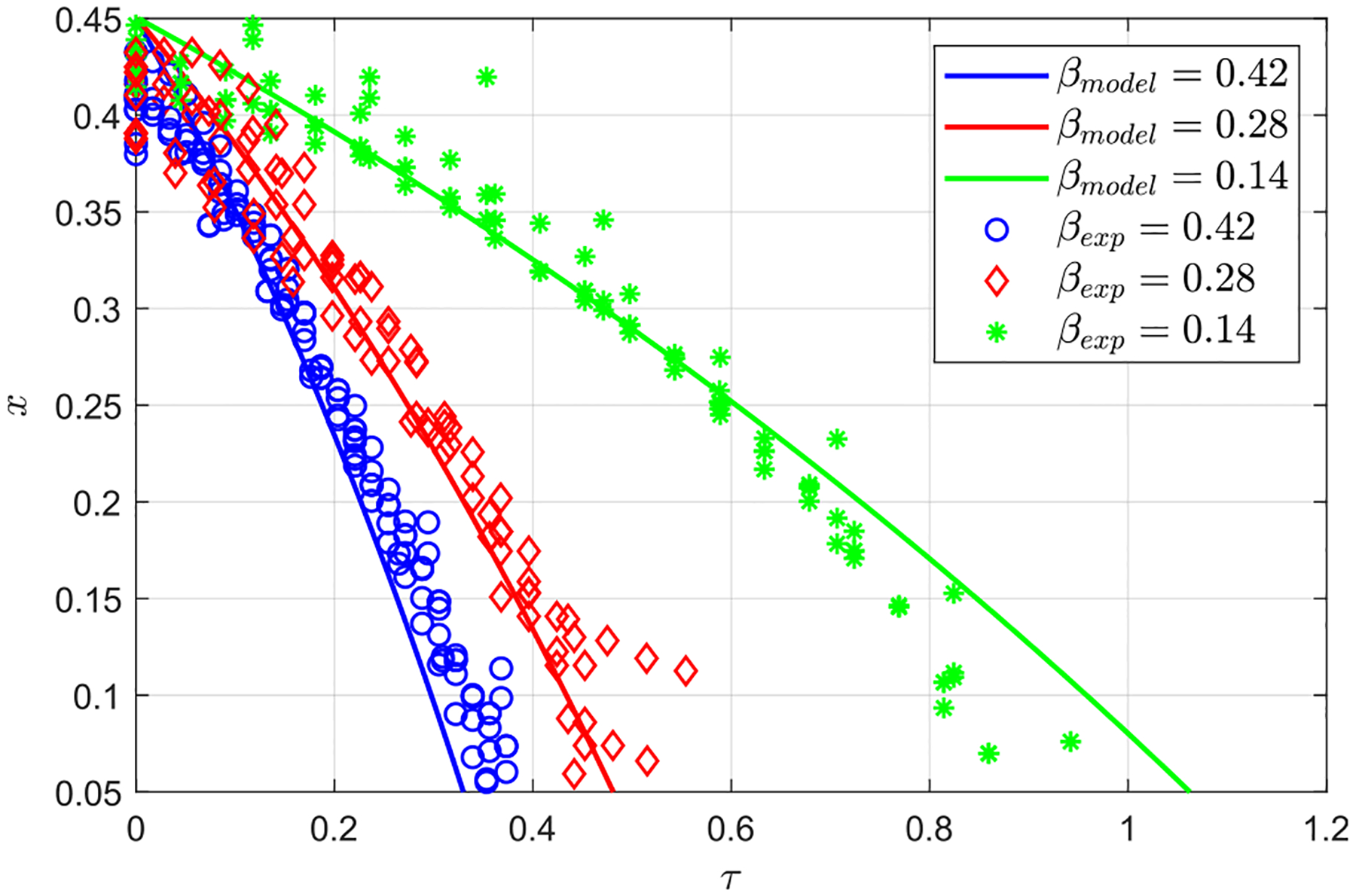
Temporal evolution of the solute along the spinal canal for α=4.39 and β=0.14,0.28,and0.42, respectively (experiments 1–6 in Table I). Symbols represent the experimental measurements, and the solid lines the results given by the theoretical model with γ=1.

To better quantify the effect of β, the time evolution of the solute front as it moves toward the canal entrance, between x=0.45 and x=0.05, is shown in Fig. 7 for β=0.14,0.28,and0.42 and a constant value of α=4.39, both for the experiments and the theoretical model. As it has been previously mentioned, in the range of eccentricities considered here, the solute rises faster as β increases. The plots indicate that the increase in the dispersion rate is not linearly proportional to β. For instance, the time needed for the solute to reach the canal entrance is halved when β increases from 0.14 to 0.28, while it is reduced by approximately only 25% when β is increased from 0.28 to 0.42. It is also of interest that the slope of the curves, -dx/dτ, increases as x decreases, indicating that the rising velocity increases as the solute approaches the entrance of the canal. All of these features are also captured by the reduced model, with results represented by solid curves in Fig. 7.

**FIG. 8.**
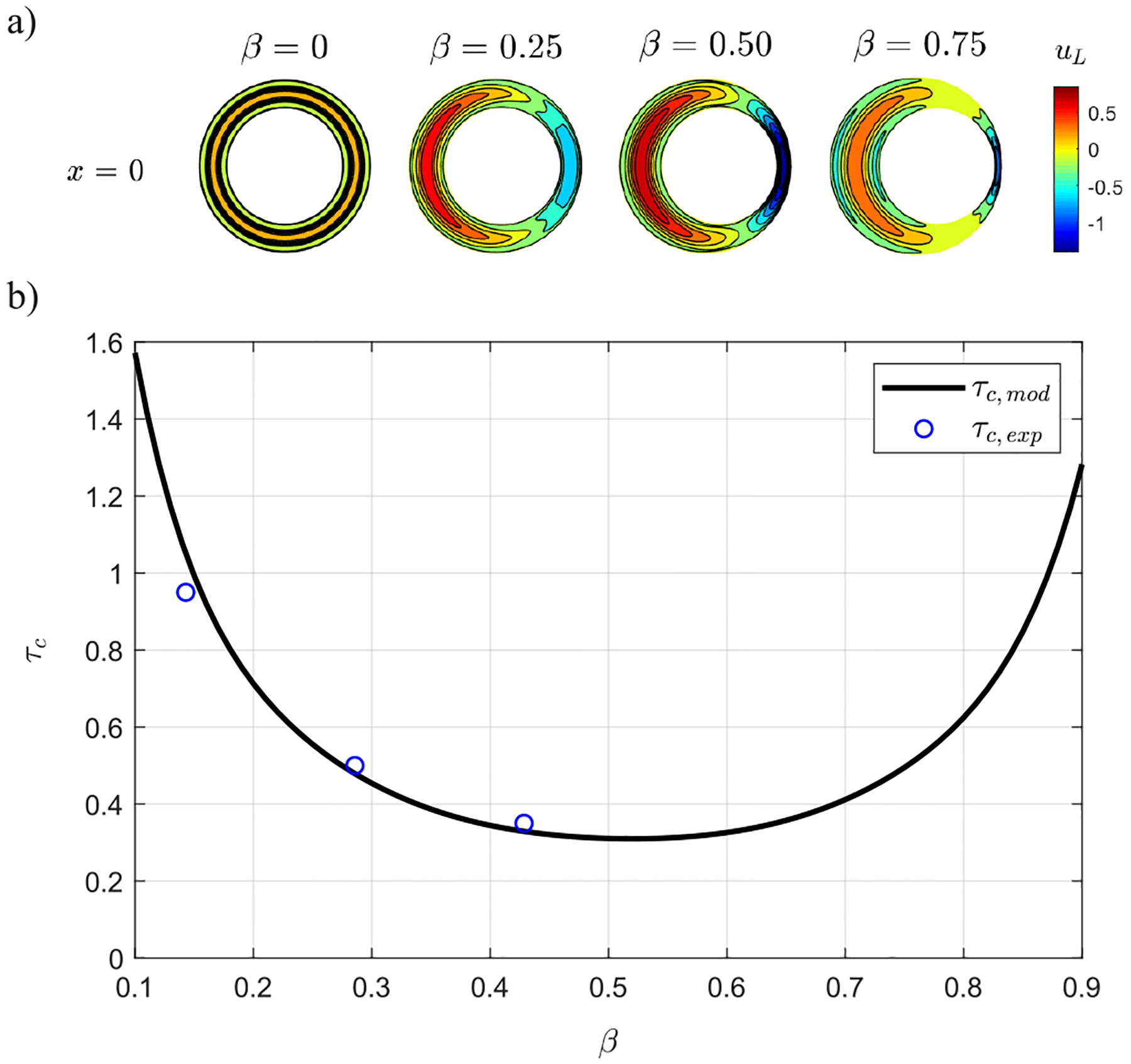
(a) Contours of the axial component of the Lagrangian velocity, $u_{L}$, at the entrance of the canal, x=0, for different values of β, provided by the theoretical model. (b) Time taken by the solute to travel from x=0.45 to x=0.05 as a function of β, defined as $\tau_{c}$. Symbols correspond to the experimental measurements, and the solid line represents the model predictions with uniform compliance y=1.

The model was used to further investigate the effects of variations of β, with results given in Fig. 8, including distributions of the axial component of the Lagrangian velocity, $u_{L}$, at the entrance of the canal, x=0, for different values of β [Fig. 8(a)], as well as the variation with β of the time that the solute takes to travel from x=0.45 to x=0.05 [Fig. 8(b)]. Peak velocities are seen to initially increase with β, reaching a maximum value at β≃0.54, for which the associated time needed to reach x=0.05 is correspondingly minimum, as shown in Fig. 8(b). For values of β≥0.54, the proximity of the walls at s=0 causes the peak velocity to decrease in magnitude, as shown for the case of β=0.75 in the contours of velocity displayed in Fig. 8(a). Similarly, the net flow rate entering/exiting the canal decreases monotonically as β increases in the range of 0≤β≤0.54.

**FIG. 9.**
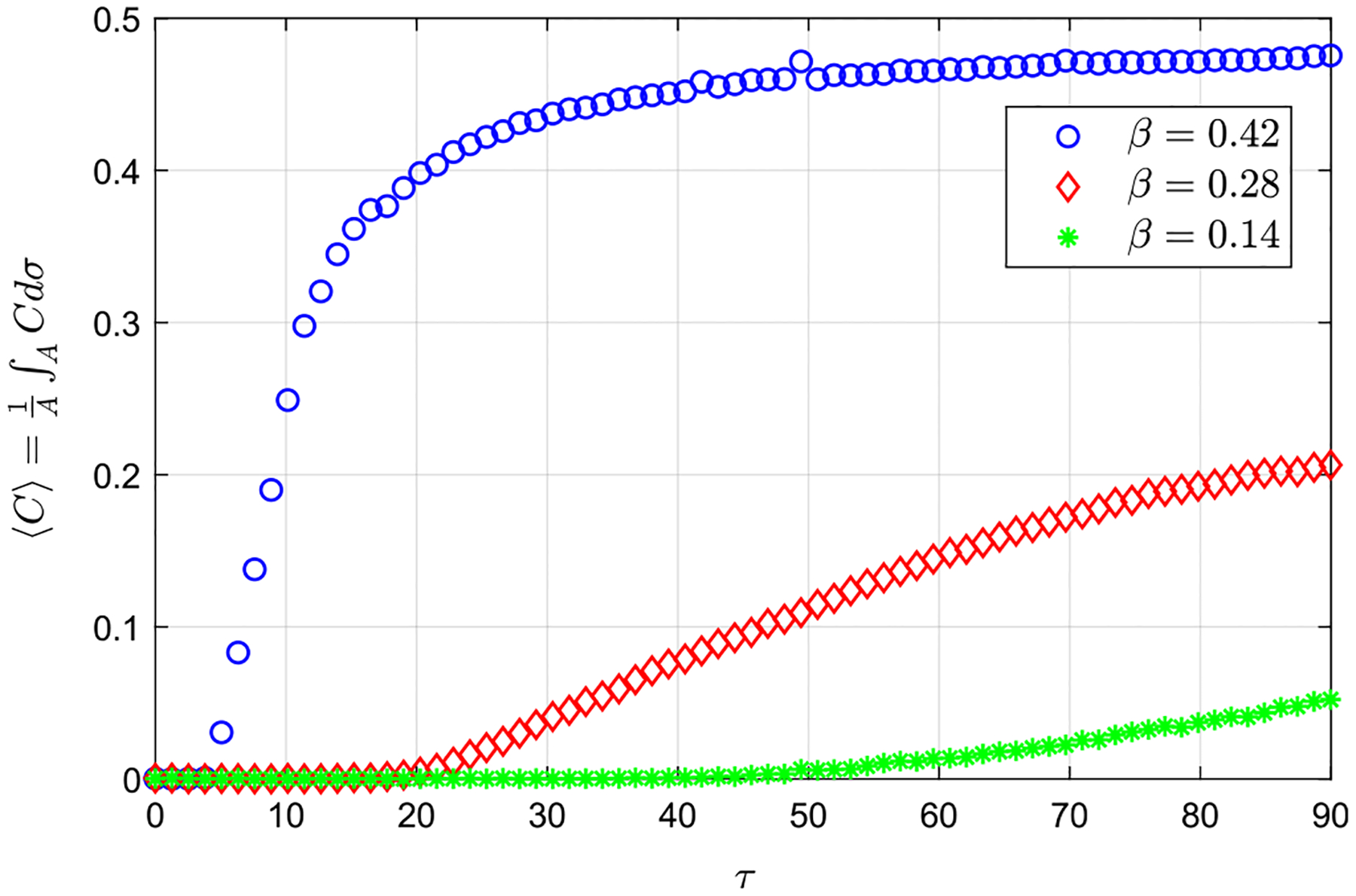
Experimental measurements of the temporal evolution of the solute concentration in the cranial vault, $\langle C\rangle (\tau )=1/A\int_{A}C\;d\sigma$, for α=4.39 and β=0.14,0.28,and0.42, respectively (experiments 7, 9, and 11 in Table I).

In addition to performing experiments to describe the transport of a solute along the canal, we also characterized the time evolution of fluorescein concentration at the cranial vault (experimental sets 7–12 in Table I). In these experiments, the temporal evolution of the light intensity emitted by the fluorescein reaching the measuring windows in the reservoir was recorded, $\langle C\rangle (\tau )=1/A\int_{A}C\;\text{d}\sigma$. Here, A is the area of the measuring windows, and C is the solute concentration, which is proportional to the light emitted by the fluorescein. Figure 9 displays the temporal evolution of the mean solute concentration in the interrogation windows in the cranial vault for β=0.14,0.28,and0.42 corresponding to α=4.39. In accordance with the results of the solute transport in the *spinal canal* described above, it can be seen that the time at which the fluorescein begins to be detected in the region of interest (ROI) increases as β decreases. Note that such time does not correspond with $\tau_{c}$ reported in Fig. 8 since the amount of fluorescein in the ROI must be larger than a given threshold to be detected in these experiments. Nevertheless, it can be observed that the rate at which the solute reaches the cranial vault increases with β, since d⟨C⟩/dτ increases with β. Interestingly, the quasi-asymptotic value of the concentration that reaches the cranial vault also increases with β. This result is attributable to the existence of increasing rising velocities at the bottom of the canal for increasing values of β. Quantitative information regarding dispersion times is important in connection with ITDD processes, especially in the case of drugs of short half-life.^48^

**FIG. 10.**
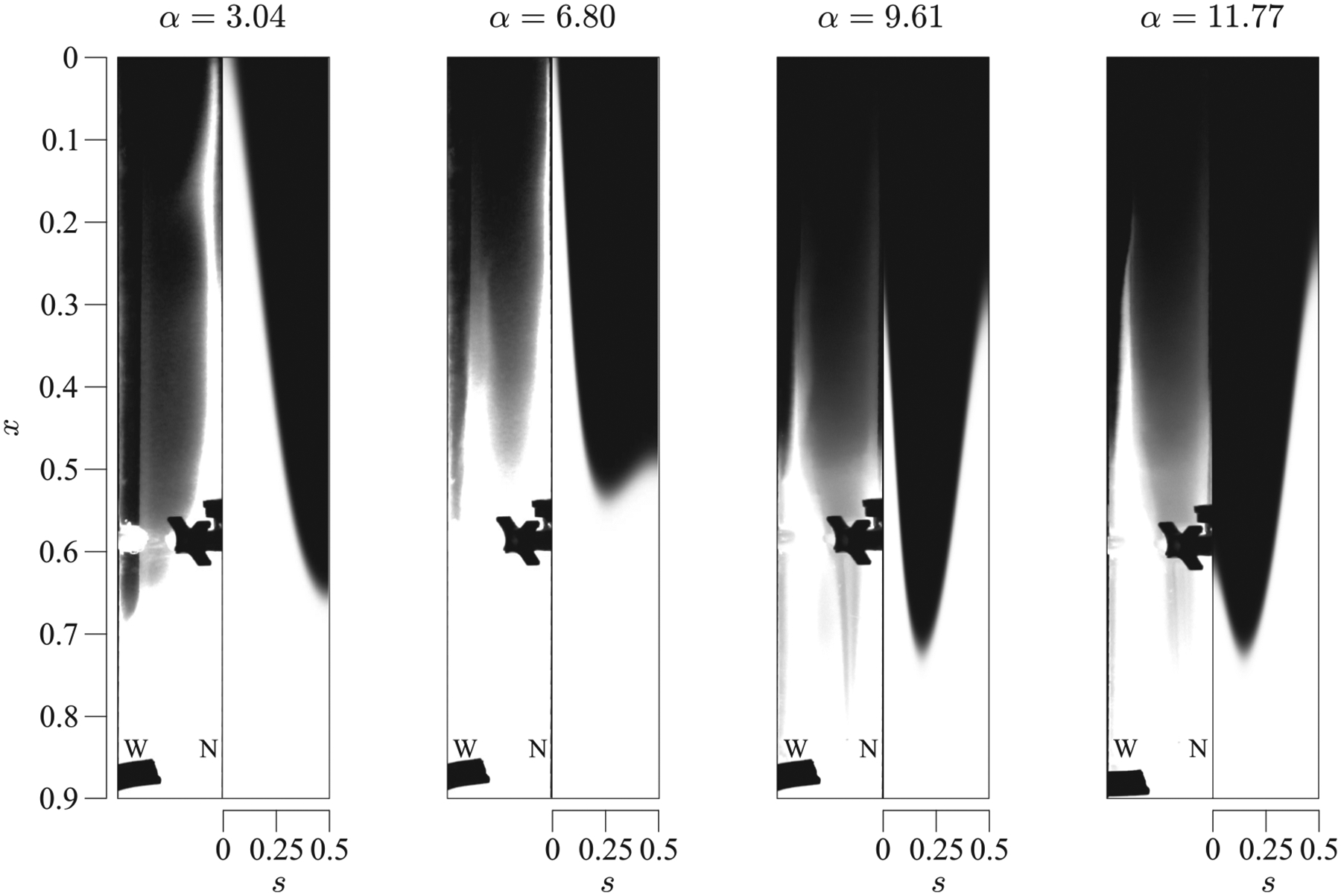
Distribution of a solute, initially filling the canal from x=0.5 to x=1, for α=3.04,6.80,9.61,and11.77, where β=0.42 in the straight configuration, corresponding to experiments 13–16 of Table I. Experimental results are represented on the left-hand side of the panels, while the theoretical predictions obtained integrating Eq. (4) are represented on the right-hand side.

The effects of the oscillating frequency were investigated in experiments with constant eccentricity β=0.42 by varying α from 3.04 to 11.77 (experimental sets 13–16 in Table I). Figure 10 shows the distribution of solute, obtained both experimentally (left-hand side of the panels), and using the theoretical model (right-hand side of the panels), at different selected times. The results reveal that variations in α lead to markedly different flow topologies and associated velocities. This strong dependence of the steady streaming on the Womersley number is well known in oscillatory flows over obstacles,^49^ but had not previously been demonstrated in connection with the flow in the spinal canal. For the lowest values of α, i.e., α=3.04 and α=4.39, the generated net flow rises up along the narrow part of the canal, s=0, along a zone that gets thinner as α increases, while it decreases along the widest region, s=0.5. However, for larger values of α, the flow also begins to rise around the widest section (see panel corresponding to α=6.80). Indeed, for intermediate values of α, i.e., α=6.80 and α=9.61, the solute rises along both the narrow and wide regions of the canal, this movement becoming more important in the wide region for increasing values of α, i.e., 9.61. Finally, for the largest value of α=11.77, the flow dynamics inverts almost completely and the solute moves upward mainly along s=0.5 (widest section). There is a good qualitative agreement between the model and the experiments, although the discrepancies are somewhat larger than those previously found when varying β for α=4.39.

**FIG. 11.**
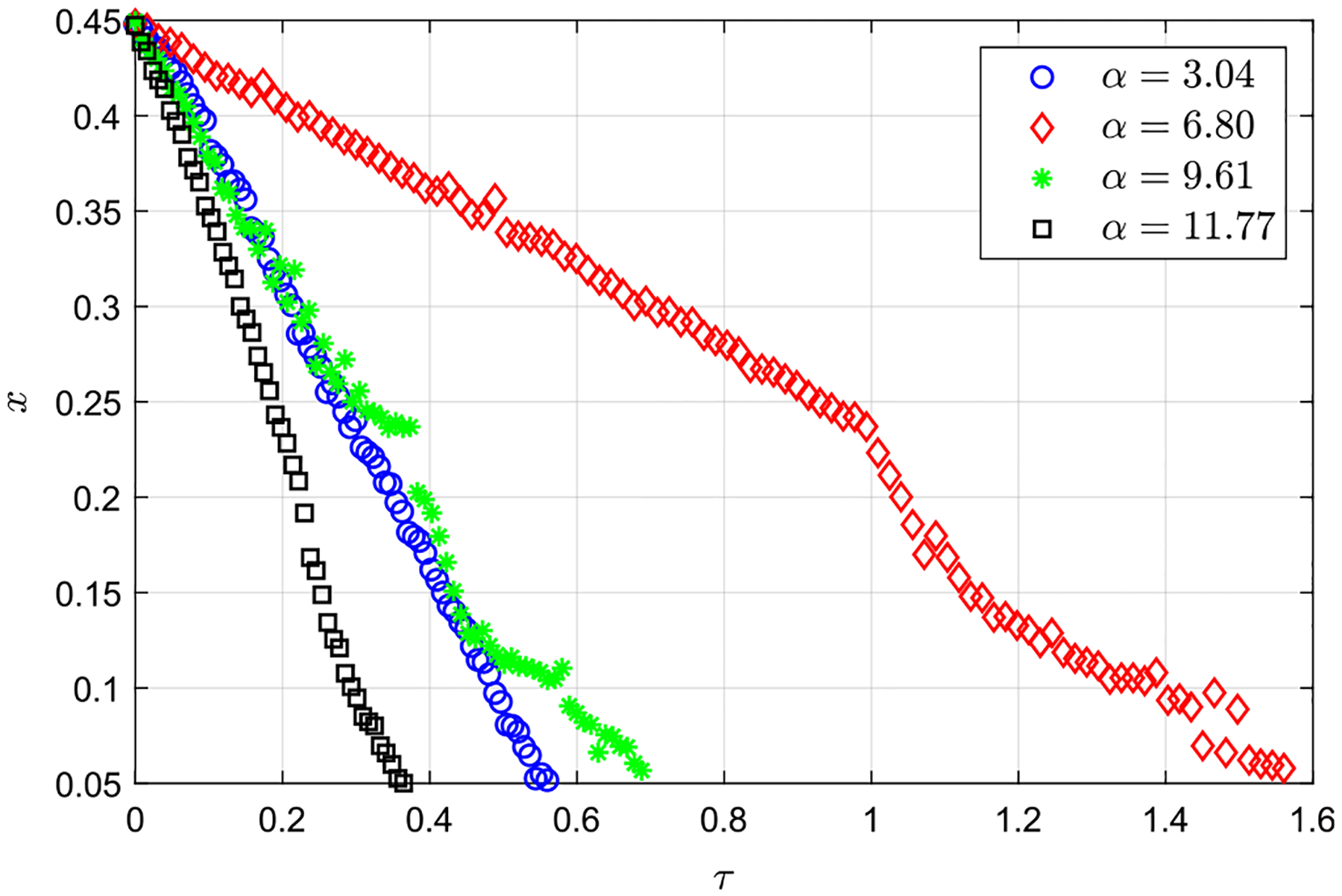
Temporal evolution of the solute along the spinal canal for β=4.2 and α=3.04,6.80and9.61and11.77, respectively (experiments 13–16 in Table I).

To experimentally quantify the effect of α on solute transport, Fig. 11 includes the temporal evolution of the uppermost front of a solute initially located at x=0.5. In all cases, the transport velocities are seen to increase at positions closer to the canal entrance. The results reveal a non-monotonic behavior in the ascending velocity of the solute for increasing values of α, in that the rising velocity displays a pronounced decrease when α is increased from 3.04 to 6.80 but increases for larger values of α, indicating that the solute transport is less efficient at intermediate values of α, i.e., 3.04≤α≤9.61.

**FIG. 12.**
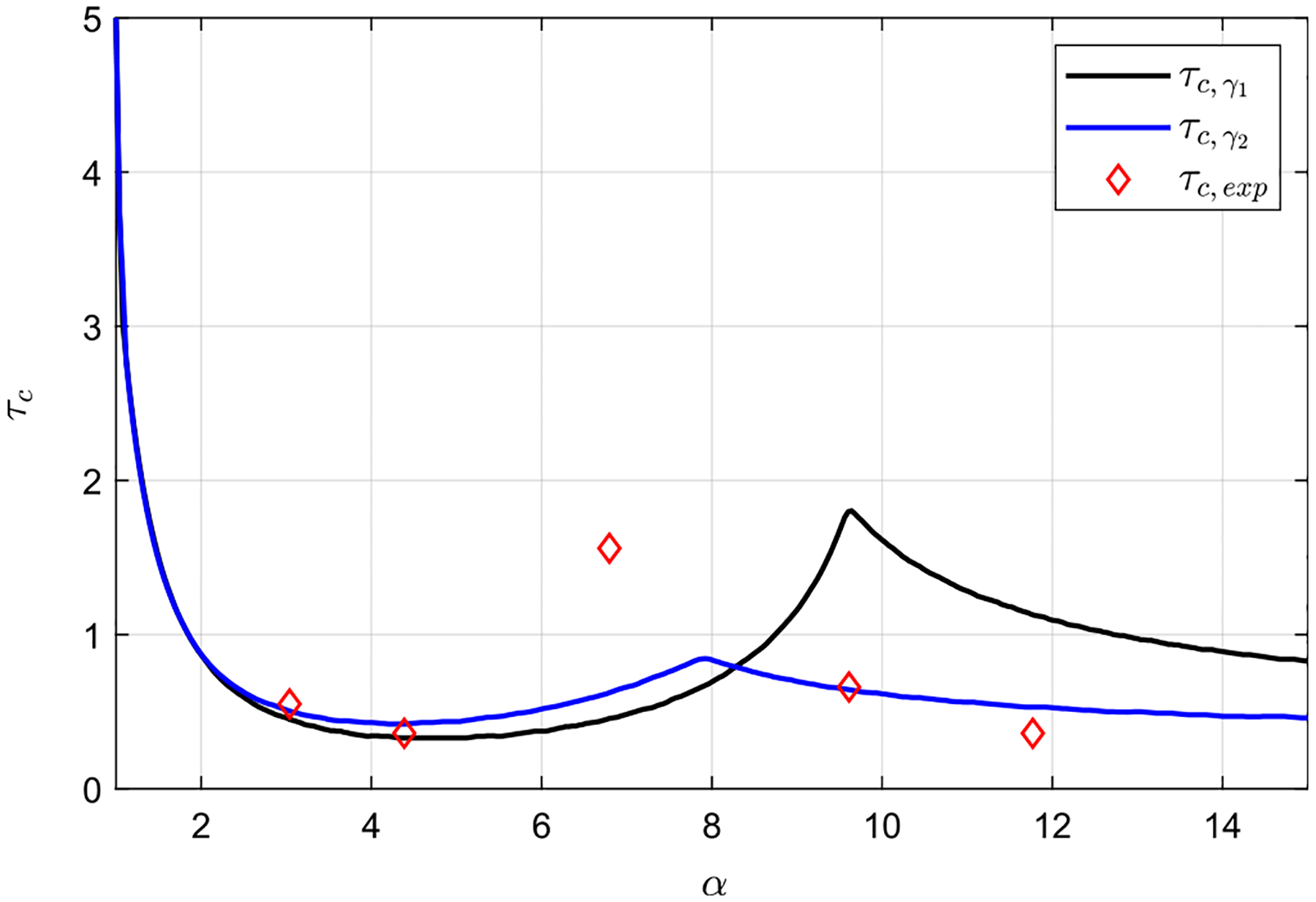
Dependence of the time taken by the solute, initially located at x=0.5, to travel from x=0.45 to $x=0.05,\tau_{c}$, with α. Symbols represent the experimental measurements, and solid lines the model predictions obtained for $\gamma_{1}=1$ and $\gamma_{2}(s)=0.2-9.6\left(2s^{2}-s\right)$, respectively. Here, β=0.42.

The influence of α is further investigated by evaluating the time $\tau_{c}$ taken by the solute, initially located at x=0.5, to travel from x=0.45 to x=0.05, with values represented in Fig. 12 as a function of α. The figure includes the experimental results, together with predictions obtained with the model using two different compliance functions, γ(x,s). First, a uniform compliance $\gamma_{1}=1$, used up until now, has been considered, for which the small deformations of the canal thickness are axisymmetric. Second, to account for possible experimental asymmetries when the compliant tube deforms, a compliance function given by $\gamma_{2}(s)=0.2-9.6\left(2s^{2}-s\right)$ was also considered, which corresponds to a canal slightly more compliant at s=±0.25 than in the symmetry plane s=0 and s=0.5. In both cases, three different regions can be identified in the evolution of $\tau_{c}$ with α. Initially, for small values of α, for which it has been observed that the flow rises through the narrowest section of the canal, $\tau_{c}$ decreases with α. However, for intermediate values of α, for which the solute rises along both the narrow and wide regions of the canal, $\tau_{c}$ increases until it reaches a maximum. Finally, for larger values of α, for which the flow rises through the widest section of the canal, $\tau_{c}$ decreases again as α increases. Interestingly, $\tau_{c}$ barely depends on the compliance factor in the range of 0<α<5, indicating that the results presented above for α=4.39 and varying values of β, where γ=1, were not affected by the function of γ(x,s) selected. However, for α>5, the differences in the values of $\tau_{c}$ obtained with the two compliance functions become more relevant. Probably, the most striking difference is the decrease in the local maximum when γ is assumed to depend on s, which takes place at lower values of α. In fact, the local maximum obtained with $\gamma_{1}=1$, corresponding to the case α=9.6, is $\tau_{c}=1.79$, while for $\gamma_{2}$, the maximum, now for α=7.9, decreases to $\tau_{c}=0.84$. The differences between the experiments and the model with uniform compliance $\gamma_{1}$ are significant, especially for α≥6. Such discrepancies could be explained by the behavior of the flexible tube used in the experiments, stemming from the difficulties to completely preserve the eccentricity, the geometric inaccuracies or the asymmetric behavior of the elastic tube deformation, represented by $\gamma_{2}$ at large α values. However, the results obtained by implementing the model with a non-uniform compliance function, $\gamma_{2}$, agree fairly well with the experimental ones, which also exhibit a local maximum of $\tau_{c}$ although at α≈8.

### B. Variable eccentricity configuration

As previously discussed, the annular cross section of the human SSAS displays an eccentricity that varies along the spinal canal, as the position of the spinal cord relative to the dura mater varies. In previous works, these variations have been postulated to play a relevant role in the dynamics of the flow of CSF. While Refs. 11 and 36 reported the formation of closed recirculating Lagrangian vortices in the canal that hampered the transport of a solute, no experimental evidence corroborating these results is currently available, thereby motivating the present analysis, which employs the configuration sketched in Fig. 2(c), where $\overset{‾}{h}\left(x,s\right)=1-\beta \;\text{cos}\left(2\pi x\right)\;\text{cos}(2\pi s)$ with β=0.5, all experiments performed with α=4.45.

**FIG. 13.**
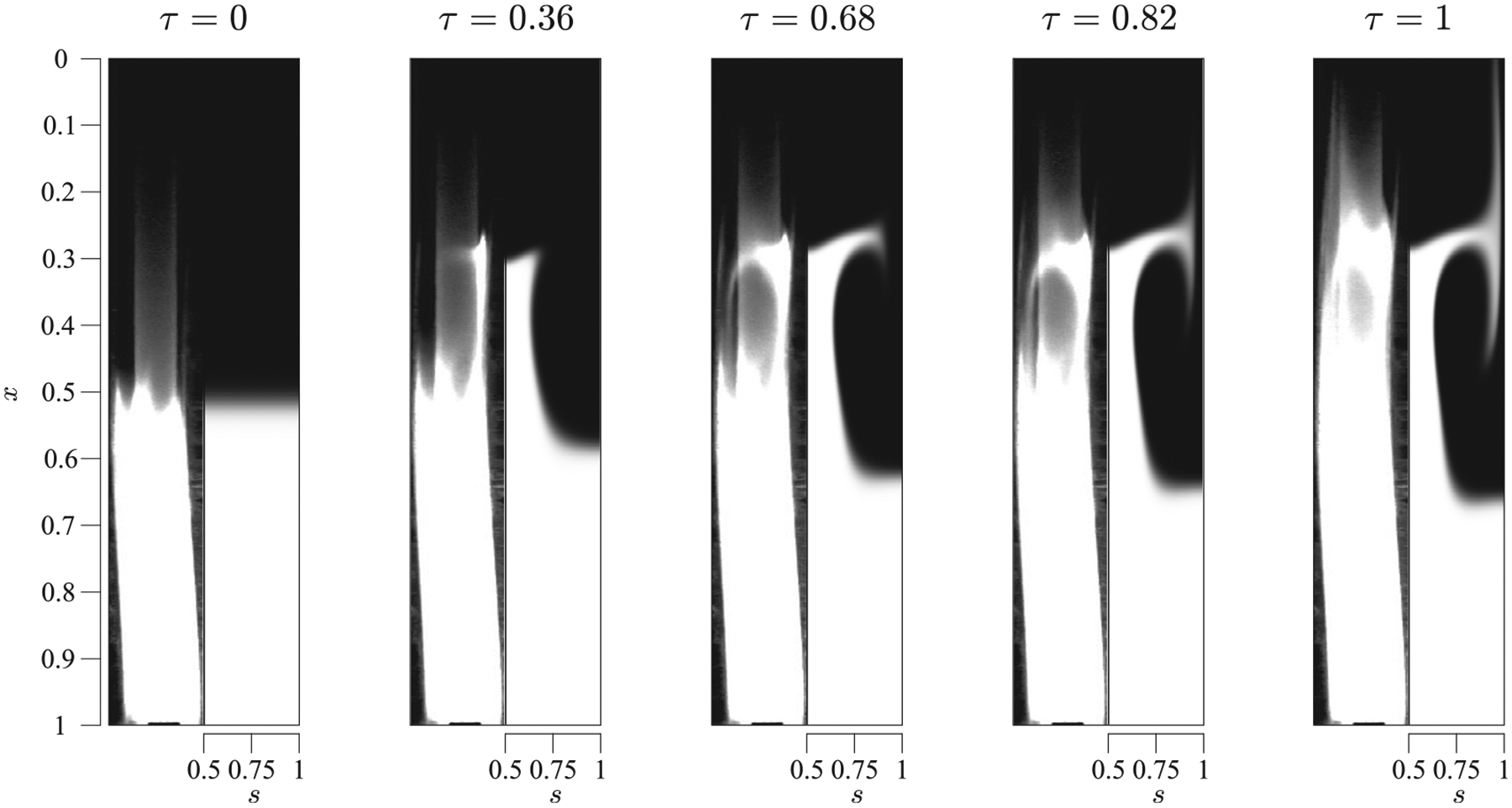
Time sequence of the evolution of a solute along a curved canal of unperturbed thickness $\overset{‾}{h}\left(x,s\right)=1-\beta \;\text{cos}\left(2\pi x\right)\;\text{cos}(2\pi s)$, with β=0.5, initially filling the canal from x=0.5 to x=1, corresponding to experiment 17 of Table I. Experimental results are represented on the left-hand side of the panels, while the theoretical predictions obtained integrating Eq. (4) are represented on the right-hand side.

As shown in Fig. 13, where the experimental/model results are shown in the left-hand/right-hand sides of each panel, for this value of α a volume of solute initially located at x≤0.5 moves upward (downward) around the narrow (wide) part of the canal. In this configuration, at x=0.5, the narrowest section corresponds to s=0.5. Focusing on the upward motion, one can observe that, as the solute reaches x≈0.27(τ≈0.36), it slows down dramatically and starts moving azimuthally from s=0.5 toward s=1 due to the change of eccentricity, since the narrow (wide) section changes from s=0.5(s=0,1) upstream from that location. This change of eccentricity provokes the formation of closed recirculating regions along the canal,^11,36^ so that, instead of continuing its progress along the canal, the solute turns around and moves down through the wide section (see panels at τ=0.68,0,82,and1 in Fig. 13). Only a small amount of solute is observed to cross the boundary between recirculating regions to rise toward the canal entrance (see the narrow filament of solute moving upward in the left side of the flexible tube in the experiments, and at s=1 at τ=1 in Fig. 13). As expected, the amount of solute reaching the cranial vault is much smaller than in the case of the straight configuration and it takes longer to arrive. The comparisons indicate that the theoretical model accurately predicts the motion of the solute in the canal.

**FIG. 14.**
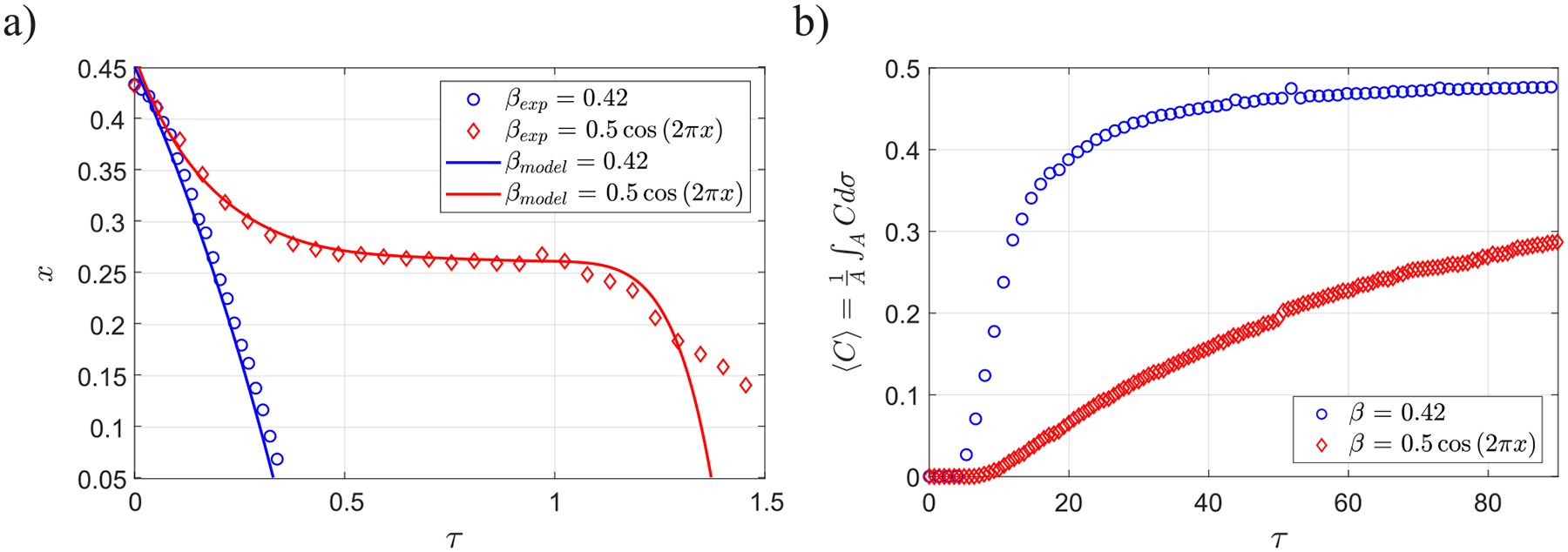
(a) Temporal evolution of the solute along a constant eccentricity canal of β=0.42 (experiment 5 in Table I), and a canal whose eccentricity varies with x as βcos(2πx), with β=0.5 (experiment 17 in Table I), for α≈4.4. Symbols represent the experimental measurements, and the solid lines the results given by the theoretical model with γ(x,s)=1. (b) Experimental measurements of the temporal evolution of the solute concentration in the cranial vault, $\langle C\rangle (\tau )=1/A\int_{A}C\;d\sigma$, for the constant and the variable eccentricity canals (experimental sets 11 and 18, respectively, in Table I).

The above results suggest that a variable eccentricity might have a major impact on the flow topology and the associated transport rate along the SSAS. To further quantify this effect, Fig. 14(a) represents the temporal evolution of the axial location of the solute front as it travels toward the canal entrance determined both experimentally and using the model for the constant and variable eccentricity configurations. It can be observed that, in both cases, the experimental results and the predictions given by the analytical model agree fairly well. Initially, for x≤0.35, the evolution is similar in both cases, i.e., increasing transport velocity at higher locations (decreasing values of x). However, differently from the constant eccentricity case, in the canal of varying eccentricity, the solute motion slows down as it approximates the stagnation point separating the upper and central recirculating regions, located at x≈0.27. At this position, where the flow turns azimuthally from s=0.5 toward s=0,1, the fluid hardly advances toward the canal entrance [see the plateau at 0.5≤τ≤1.2 in Fig. 14(a)], to subsequently move upward again in that region, which is now the narrow one, with increasing velocities at locations closer to the cranial vault. As a consequence, the time for the solute to reach the canal entrance, $\tau_{c}$, becomes three times larger than that in the constant eccentricity configuration for the case at hand. Furthermore, not only the advance velocity but also the amount of solute able to reach the entrance of the canal also decreases, since a part of the solute remains trapped in the lower recirculating Lagrangian vortex located below x≈0.27.

Figure 14(b) displays the comparison of the time evolution of solute concentration in the cranial vault for the constant and variable eccentricity cases. Note that the rate at which the solute reaches the cranial vault is lower in the case of variable eccentricity, since d⟨C⟩/dτ is smaller in this case than that in the canal with constant eccentricity, as well as the quasi-asymptotic value of the concentration that reaches the cranial vault. In view of the above results, it is evident that the formation of recirculating flow patterns hinders the dispersion of the solute, with important implications concerning the rate at which a drug injected in the lumbar region can reach target locations at the cervical or cerebral level.

## V. CONCLUSIONS

This experimental analysis addresses the motion of CSF in the human SSAS, with attention given to the dispersion rate of a solute injected at the low thoracic and lumbar regions, a key process in connection with ITDD procedures. To that aim, we have conducted *in vitro* experiments in a simplified annular geometry modeling the SSAS, similar to that considered in previous theoretical^19,28^ and numerical^36^ studies. A programmable pump has been used to induce a harmonic fluid motion in and out of the compliant canal with a stroke volume ΔV much smaller than the total volume contained in the canal, that being the relevant limiting case for the flow in the SSAS. The solute motion has been characterized with the use of LIF techniques. In particular, the effects of the canal eccentricity and the oscillation frequency, the latter measured by the Womersley number, have been assessed in a configuration with uniform eccentricity. A modified geometry allowing for the spatial variation of the cross section eccentricity, a key feature of the human spinal canal, has also been considered. The results have been compared with predictions obtained using the analytical model developed in Refs. 19 and 28.

The results corroborate the existence of a slow net transport rate, with characteristic times of order $\varepsilon^{-2}\omega^{-1}\gg \omega^{-1}$, which is regulated by the convective transport driven by the time-averaged Lagrangian velocity (i.e., the sum of the Eulerian steady-streaming velocity and the Stokes-drift velocity) and, to a lesser extent, by the action of molecular diffusion across the SSAS.^28,36^ In the constant-eccentricity configuration, variations of the eccentricity are seen to yield significant variations in the induced transport velocities and associated amounts of solute reaching the canal entrance, with the solute transport achieving peak rates for intermediate values of β~0.5. Variations in the Womersley number are seen to result in important changes in the flow topology, in addition to the flow velocities. In particular, the motion of solute was found to occur toward the cranial vault (bottom) in the narrow (wide) region of the canal for β=0.42 and α≈3, whereas it occurred in the opposite direction for β=0.42 and α≈11. On the other hand, the measurements conducted using a variable eccentricity configuration revealed, for the first time in *in vitro* experiments, the formation of recirculatory Lagrangian cells along the canal. As a consequence, a part of the solute remained trapped inside these cells, thus hindering its transport toward the cranial vault. The results of this study are important in guiding future developments of predictive tools to assist clinicians and to evaluate the effectiveness of ITDD processes.

## Figures and Tables

**TABLE I.** Experimental conditions of the different sets of experiments performed. Here, β is the relative eccentricity, f is the oscillatory frequency of the motion, with $\omega =2\pi f,\alpha ={\left(h_{c}^{*2}\omega /\nu \right)}^{1/2}$ is the Womersley number, ΔV/V≪1 is the ratio between the stroke volume and the total volume in the canal, and $f_{s}$ is the data acquisition frequency.

Experiment	β	f(Hz)	ω(rad/s)	α	ΔV/V	$f_{s}(\text{Hz})$	Configuration	Type
1	0.14	0.25	1.571	4.39	0.052	0.031 25	Straight	SSAS
2	0.14	0.25	1.571	4.39	0.084	0.031 25	Straight	SSAS
3	0.28	0.25	1.571	4.39	0.052	0.050 00	Straight	SSAS
4	0.28	0.25	1.571	4.39	0.084	0.050 00	Straight	SSAS
5	0.42	0.25	1.571	4.39	0.052	0.083 33	Straight	SSAS
6	0.42	0.25	1.571	4.39	0.084	0.083 33	Straight	SSAS
7	0.14	0.25	1.571	4.39	0.052	0.008 33	Straight	Cranial vault
8	0.14	0.25	1.571	4.39	0.084	0.008 33	Straight	Cranial vault
9	0.28	0.25	1.571	4.39	0.052	0.008 33	Straight	Cranial vault
10	0.28	0.25	1.571	4.39	0.084	0.008 33	Straight	Cranial vault
11	0.42	0.25	1.571	4.39	0.052	0.008 33	Straight	Cranial vault
12	0.42	0.25	1.571	4.39	0.084	0.008 33	Straight	Cranial vault
13	0.42	0.12	0.754	3.04	0.030	0.040 00	Straight	SSAS
14	0.42	0.60	3.77	6.80	0.030	0.100 00	Straight	SSAS
15	0.42	1.20	7.54	9.61	0.024	0.200 00	Straight	SSAS
16	0.42	1.80	11.31	11.77	0.018	0.200 00	Straight	SSAS
17	0.50	0.35	2.20	4.45	0.052	0.017 50	Curved	SSAS
18	0.50	0.35	2.20	4.45	0.052	0.008 33	Curved	Cranial vault

## Data Availability

The data that support the findings of this study are available from the corresponding author upon reasonable request.

## NOMENCLATURE

A: Operating window area
c: Characteristic value
C: Solute concentration
$e,e_{v}$: Eccentricity: distance between internal and externa walls of the canal (constant configuration, variable configuration)
$E_{c}$: Characteristic value of the Young modulus of the experimental flexible tube
f: Dimensional frequency of the oscillatory motion
$f_{s}$: Sampling frequency of the experimental image acquisition
$h,\overset{‾}{h},h^{\prime }$: Width of the canal (temporal, unperturbed, and time-dependent radial deformation)
$h_{s}$: Width of the interior experimental flexible tube
k: Dimensionless wave number
L: Lagrangian component
L: Total length of the canal
ℓ: Perimeter of the internal surface of the canal
$p^{\prime },\overset{ˆ}{p}$: Differential pressure (axial, azimuthal)
Q: Experimental flow rate of the pump
$q_{L}$: Width-averaged Lagrangian velocity magnitude
$R_{e}$: Internal radius of the experimental plexiglass tube
$R_{i}$: External radius of the experimental flexible tube
S: Schmidt number
SD: Stokes drift component
SS: Steady streaming component
T: Dimensional period of the oscillatory motion
t: Time
$T_{s}$: Tensile strength of the experimental flexible tube
$\left(u,v,w\right)$: Curvilinear velocity components of CSF motion (axial, radial, and azimuthal)
V: Total volume of the CSF in the SSAS
(x,η,s): Non-dimensional normalized curvilinear spatial components (axial, radial, and azimuthal)
0: Leading order component
α: Womersley number
β: Relative eccentricity of the canal
γ: Compliance of the dura membrane
ΔV: Stroke volume of the CSF in the SSAS
ΔL: Stroke length
$(\Delta p{)}_{c}$: Intracranial pressure
$\delta h^{*}$: Wall deformation
$\delta p^{*}$: Pressure fluctuation
ε: Dimensionless small parameter
κ: Molecular diffusivity of the passive scalar
λ: Tracer emission wave length
ν: Kinematic viscosity of the CSF
ρ: Density of the CSF
τ: Dimensionless long-term-scaled time
$\tau_{c}$: Dimensionless arriving long-scaled time
ϕ: Fluid property
ω: Angular frequency of the CSF motion
*: Any variable denoted with upper asterisk * represents a dimensional variable
